# Supplementary material for: Epigenetic Manipulation Induced Production of Immunosuppressive Chromones and Cytochalasins from the Mangrove Endophytic Fungus Phomopsis asparagi DHS-48
Source: Mar Drugs. 2022 Sep 29;20(10):616. doi: 10.3390/md20100616 (PMC9605342; doi:10.3390/md20100616)
Supplement: Supplementary file 1 [file marinedrugs-20-00616-s001.zip › marinedrugs-1935074-supplementary.pdf]

Epigenetic Manipulation Induced Production of Immunosuppressive Chromones and Cytochalasins from the Mangrove Endophytic Fungus *Phomopsis asparagi* DHS-48

Ting Feng, † Chengwen Wei, † Xiaolin Deng, Dandan Chen, and Jing Xu*

Collaborative Innovation Center of Ecological Civilization, School of Chemical Engineering and Technology, Hainan University, Haikou 570228, P. R. China.

† These authors have contributed equally to this work.

* To whom correspondence should be addressed.

Prof. Dr. Jing Xu, Tel.: ++86–898–6627–9226, Fax: ++86–898–6627–9010, E–mail: happyjing3@163.com

Contents

Figure S1. ^{1}H-NMR of phaseolorin J (**1**) ---------- 4

Figure S2. ^{13}C-NMR of phaseolorin J (**1**)---------- 4

Figure S3. DEPT of phaseolorin J (**1**) ---------- 5

Figure S4. ^{1}H-^{1}H COSY of phaseolorin J (**1**) ---------- 5

Figure S5. HSQC of phaseolorin J (**1**) ---------- 6

Figure S6. HMBC of phaseolorin J (**1**)---------- 6

Figure S7. NOSEY of phaseolorin J (**1**)---------- 7

Figure S8. HR-ESI-MS of phaseolorin J (**1**)---------- 7

Figure S9. ^{1}H-NMR of phomoparagin D (**5**)---------- 8

Figure S10. ^{13}C-NMR of phomoparagin D (**5**)---------- 8

Figure S11. DEPT of phomoparagin D (**5**) ---------- 9

Figure S12. ^{1}H-^{1}H COSY of phomoparagin D (**5**)---------- 9

Figure S13. HSQC of phomoparagin D (**5**) ----------10

Figure S14. HMBC of phomoparagin D (**5**)----------10

Figure S15. NOSEY of phomoparagin D (**5**)----------11

Figure S16. HR-ESI-MS of phomoparagin D (**5**)----------11

Figure S17. ^{1}H-NMR of phaseolorin D (**2**) ----------12

Figure S18. ^{13}C-NMR of phaseolorin D (**2**)----------12

Figure S19. HR-ESI-MS of phaseolorin D (**2**)----------13

Figure S20. ^{1}H-NMR of chaetochromone B (**3**)----------13

Figure S21. ^{13}C-NMR of chaetochromone B (**3**) ----------14

Figure S22. HR-ESI-MS of chaetochromone B (**3**) ----------14

Figure S23. ^{1}H-NMR of pleosporalin D (**4**) ----------15

Figure S24. ^{13}C-NMR of pleosporalin D (**4**)----------15

Figure S25. HR-ESI-MS of pleosporalin D (**4**)----------16

Figure S26. ^{1}H-NMR of cytochalasin J (**6**)----------16

Figure S27. ^{13}C-NMR of cytochalasin J (**6**) ----------17

Figure S28. HR-ESI-MS of cytochalasin J (**6**) ----------17

Figure S29. ^{1}H-NMR of cytochalasin J_1 (**7**)----------18

Figure S30. ^{13}C-NMR of cytochalasin J_1 (**7**) ----------18

Figure S31. HR-ESI-MS of cytochalasin J_1 (**7**) ----------19

Figure S32. ^{1}H-NMR of cytochalasin H (**8**) ----------19

Figure S33. ^{13}C-NMR of cytochalasin H (**8**)----------20

Figure S34. HR-ESI-MS of cytochalasin H (**8**)----------20

Figure S35. ^{1}H-NMR of cytochalasin J_2 (**9**) ----------21

Figure S36. ^{13}C-NMR of cytochalasin J_2 (**9**) --21
Figure S37. HR-ESI-MS of cytochalasin J_2 (**9**) --22
Figure S38. ^{1}H-NMR of cytochalasin J_3 (**10**)--22
Figure S39. ^{13}C-NMR of cytochalasin J_3 (**10**) ---23
Figure S40. HR-ESI-MS of cytochalasin J_3 (**10**) --23
Figure S41. ^{1}H-NMR of phomopchalasin D (**11**)--24
Figure S42. ^{13}C-NMR of phomopchalasin D (**11**) ---24
Figure S43. HR-ESI-MS of phomopchalasin D (**11**) --25
Figure S44. Overlay of HPLC profiles of EtOAc extracts of *Phomopsis asparagi* DHS-48 cultivated in PDA treated with different epigenetic agents. ---26
Figure S45. HPLC spectrum for the purity of tested compounds -----------------------------------27
Table S1. Gibbs free energies[a] and equilibrium populations[b] of low-energy conformers of phaseolorin J (**1**)-- 33
Table S2. Cartesian coordinates for the low-energy reoptimized MMFF conformers of phaseolorin J (**1**) at B3LYP/6-31G(d,p) level of theory in gas--33
Table S3. Gibbs free energies[a] and equilibrium populations[b] of low-energy conformers of phomoparagin D (**5**)--- 36
Table S4. Cartesian coordinates for the low-energy reoptimized MMFF conformers of phomoparagin D (**5**) at B3LYP/6-31G(d,p) level of theory in gas-------------------------------------36
Table S5. The calculated ^{13}C NMR data for isomers of phaseolorin J (**1**) --------------------------43
Table S6. DFT-optimized structures and thermodynamic parameters for low-energy conformers of **1**--- 44
Table S7. DFT-optimized structures and thermodynamic parameters for low-energy conformers of 8-*epi*-**1**--- 45
Table S8. Optimized Z-matrixes of **1** in the gas phase (Å) at B3LYP/6-31G(d) level------------46
Table S9. Optimized Z-matrixes of 8-*epi*-**1** in the gas phase (Å) at B3LYP/6-31G(d) level------47

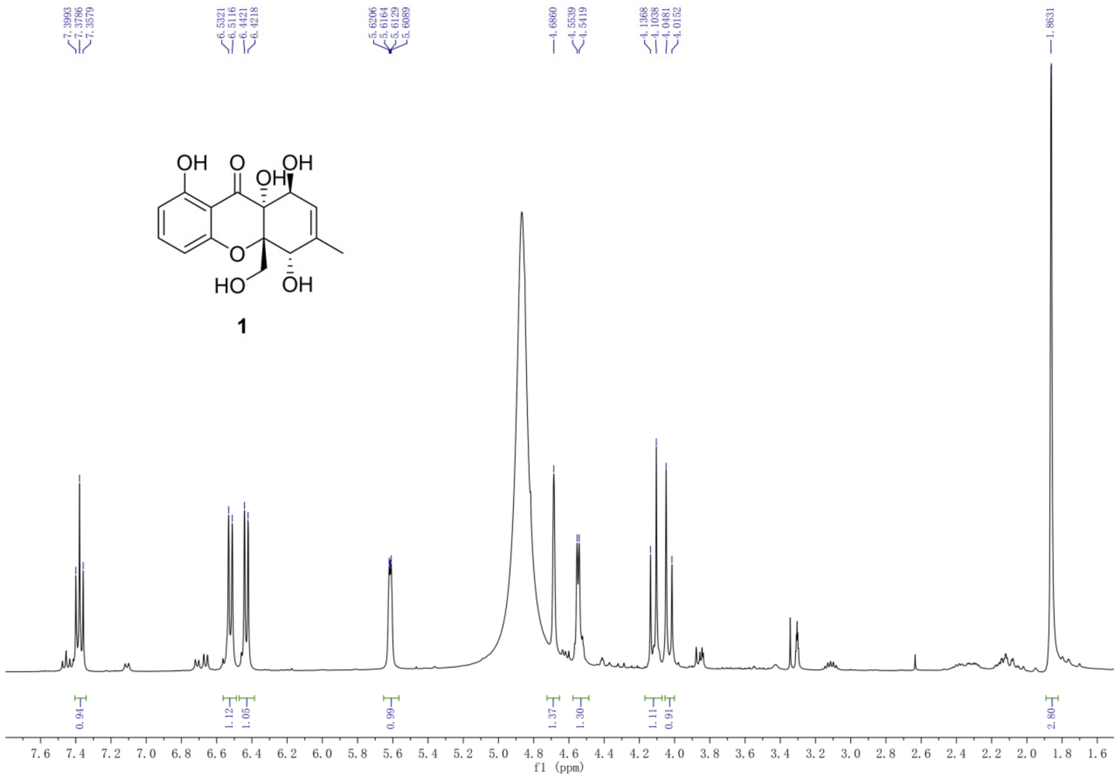

Figure S1. ^{1}H-NMR of phaseolorin J (**1**).

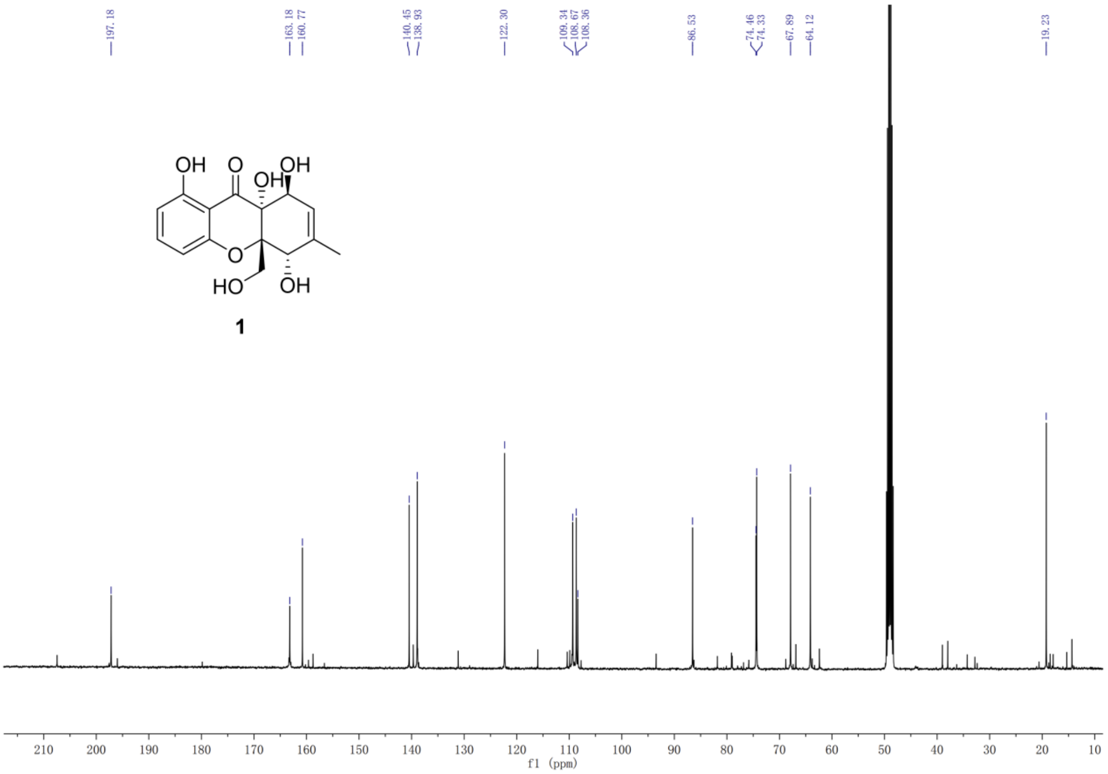

Figure S2. ^{13}C-NMR of phaseolorin J (**1**).

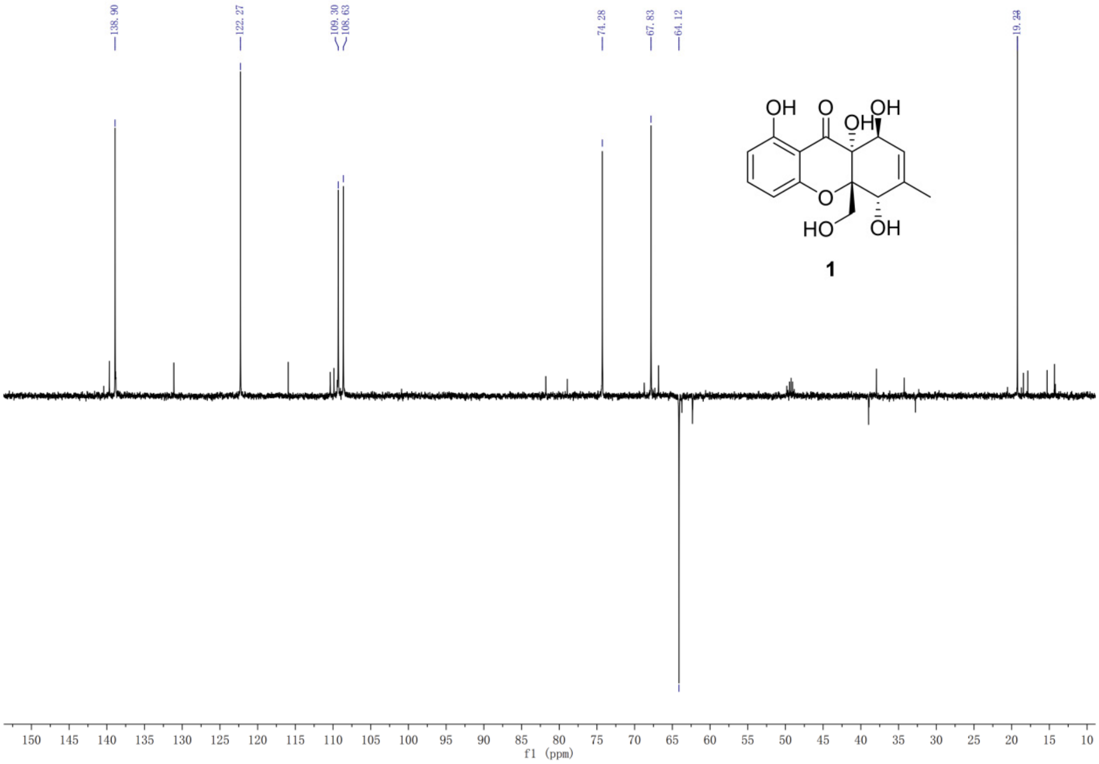

Figure S3. DEPT of phaseolorin J (**1**).

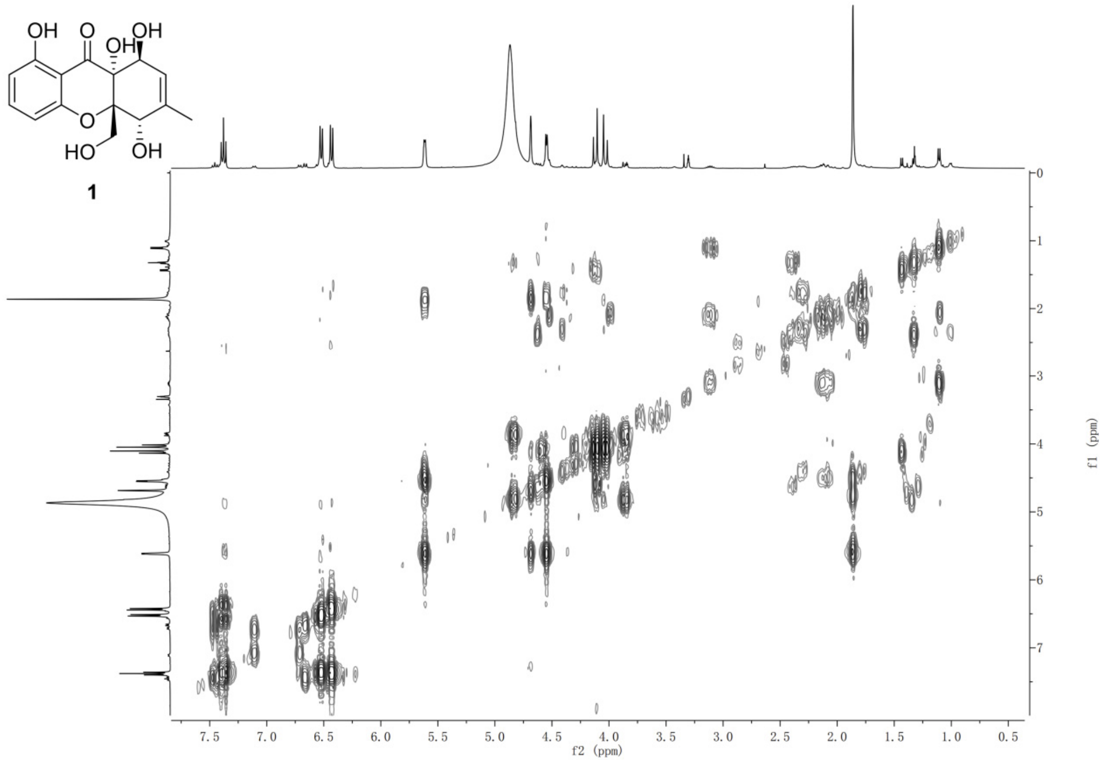

Figure S4. ^{1}H-^{1}H COSY of phaseolorin J (**1**).

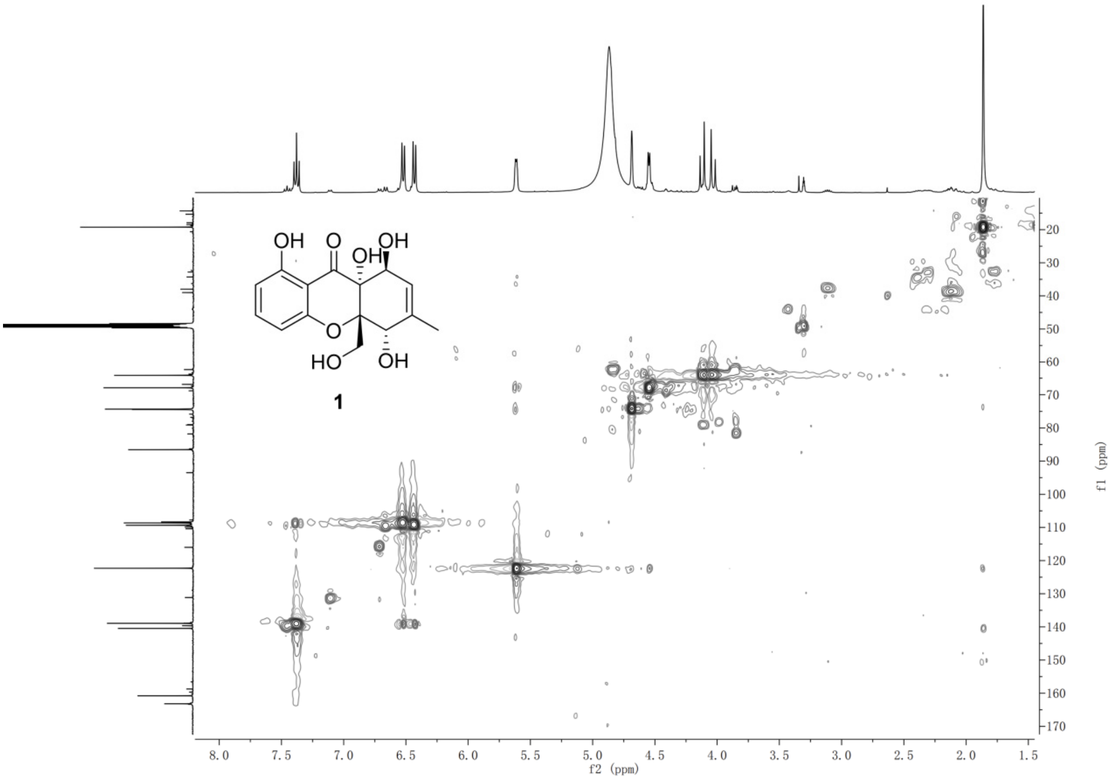

Figure S5. HSQC of phaseolorin J (**1**).

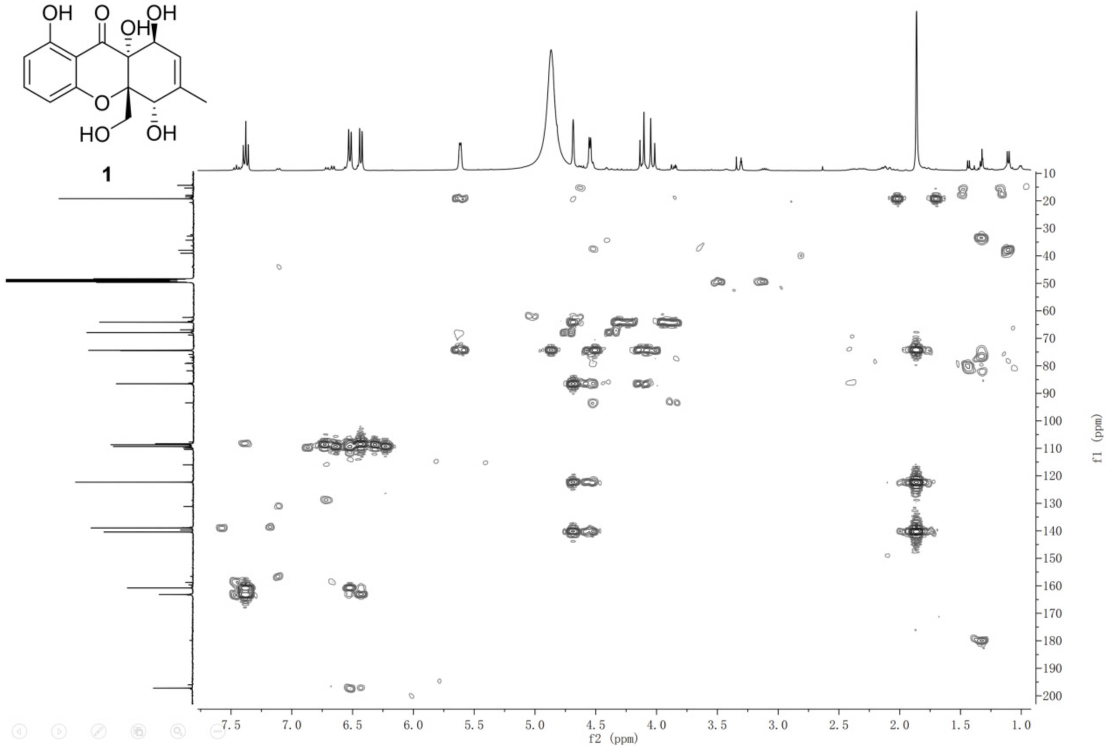

Figure S6. HMBC of phaseolorin J (**1**).

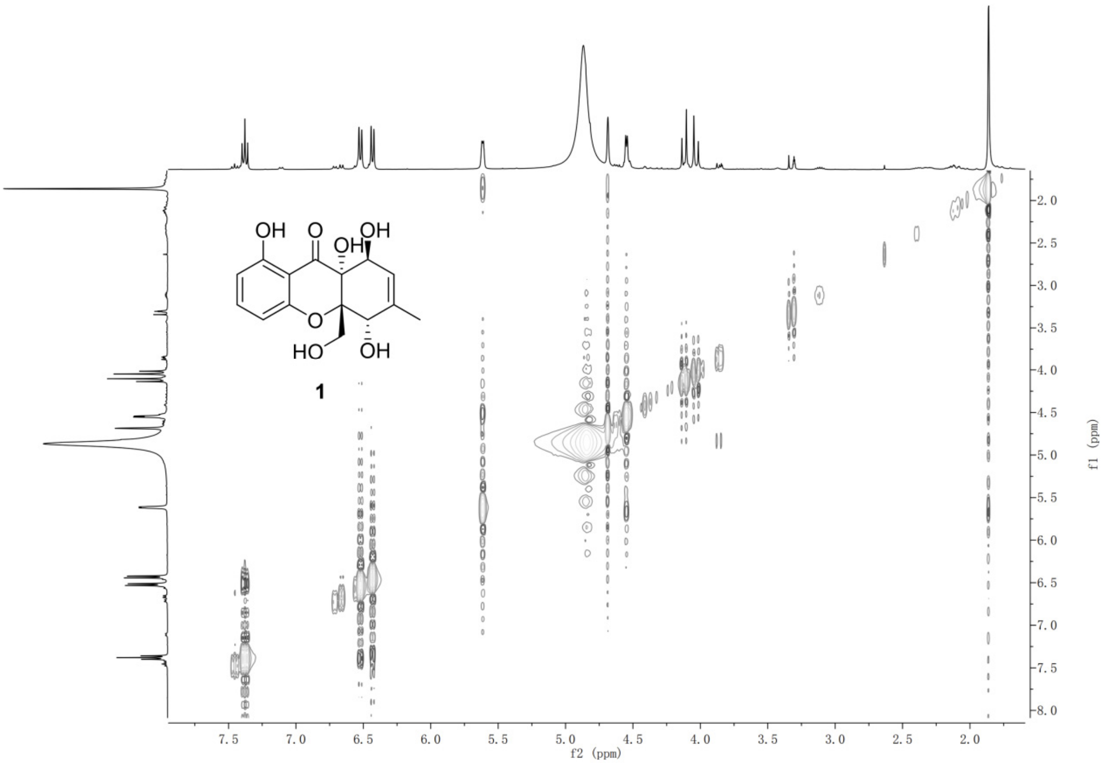

Figure S7. NOSEY of phaseolorin J (**1**).

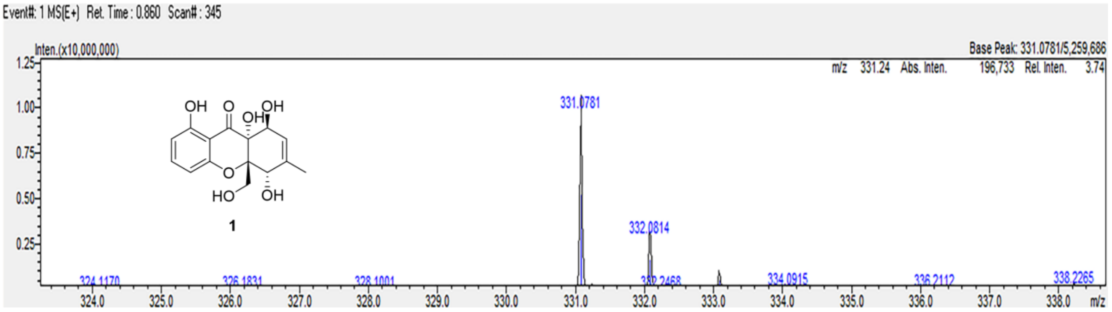

Figure S8. HR-ESI-MS of phaseolorin J (**1**).

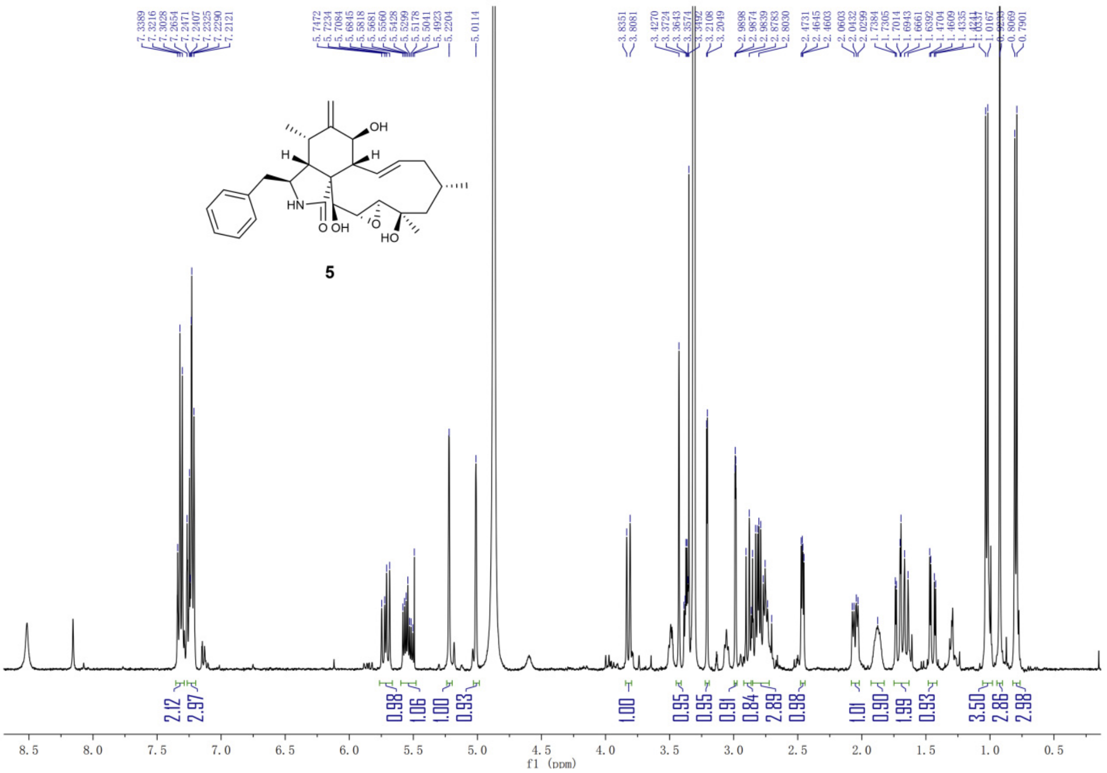

Figure S9. ^{1}H-NMR of phomoparagin D (**5**).

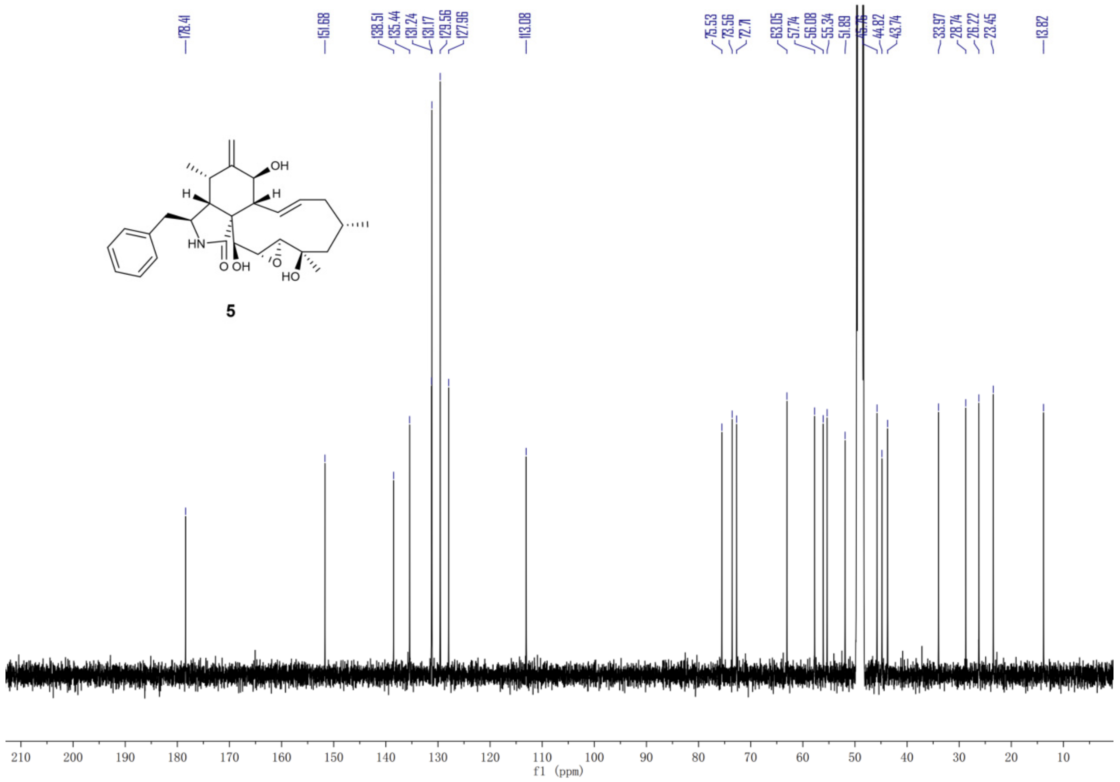

Figure S10. ^{13}C-NMR of phomoparagin D (**5**).

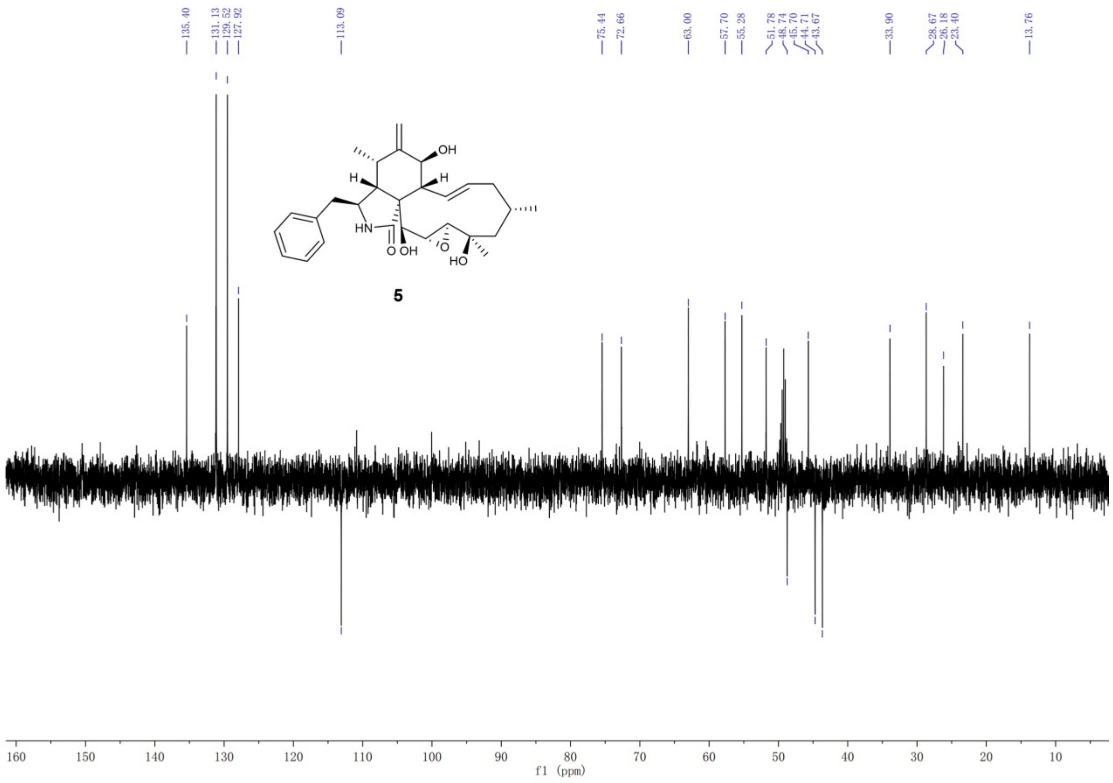

Figure S11. DEPT of phomoparagin D (**5**).

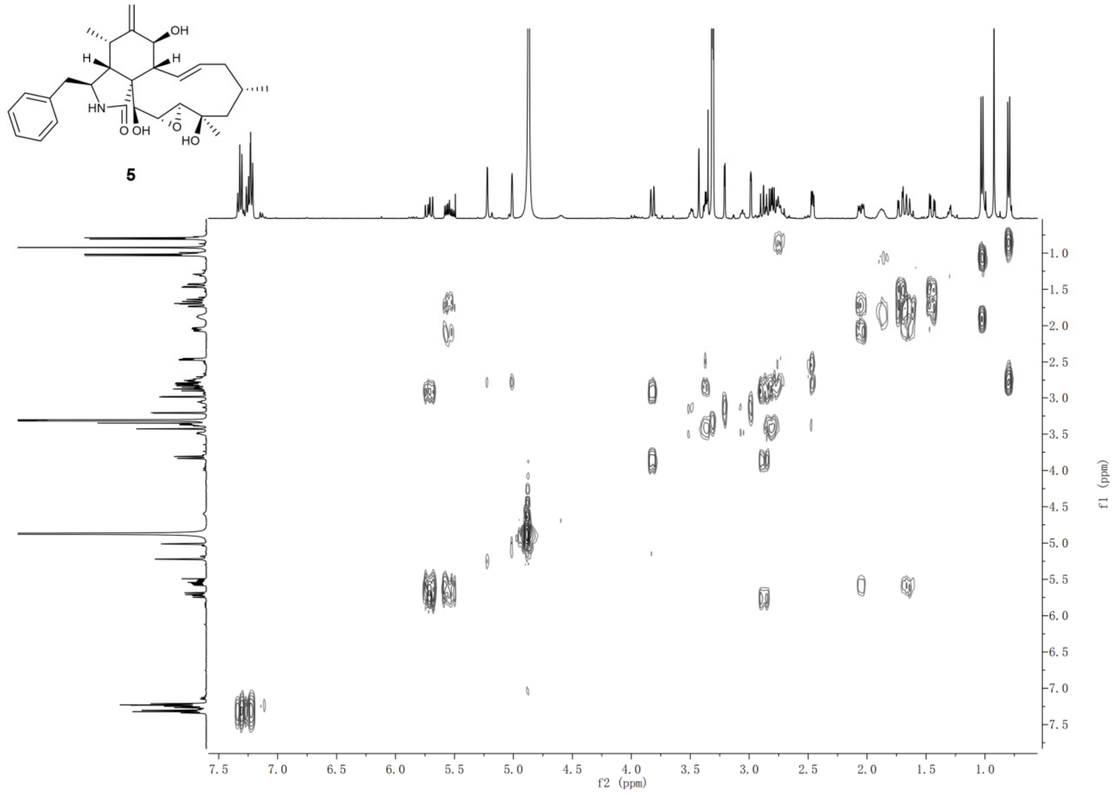

Figure S12. ^{1}H-^{1}H COSY of phomoparagin D (**5**).

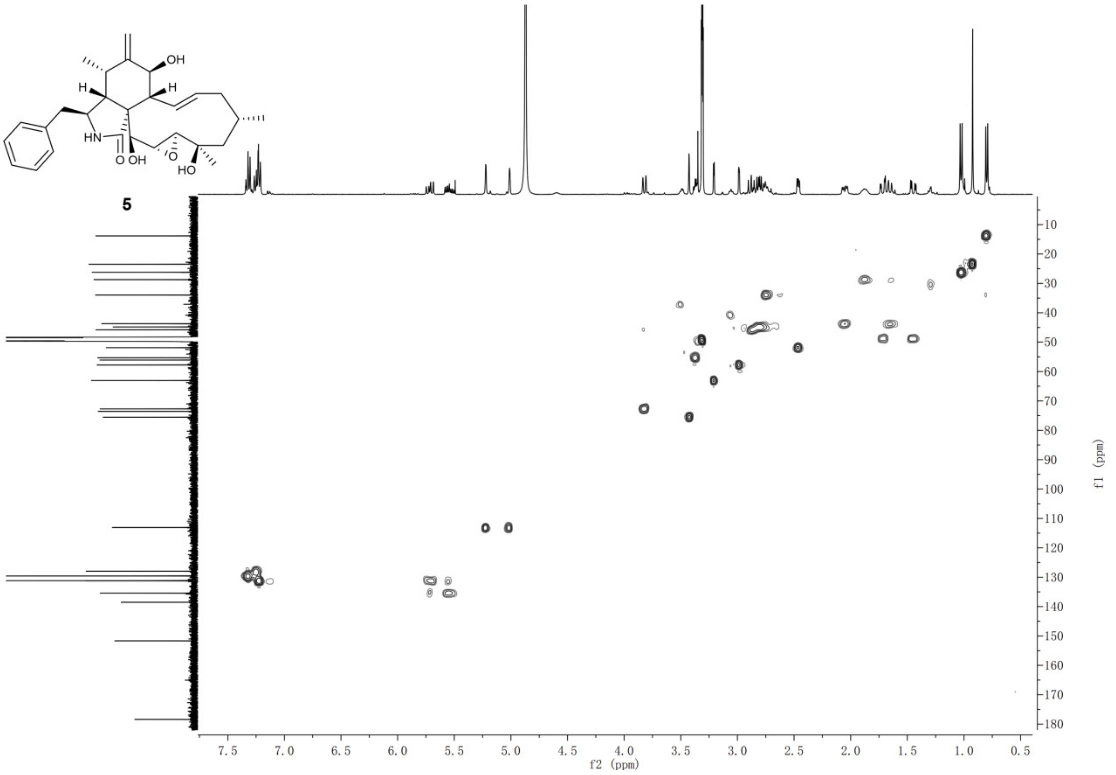

Figure S13. HSQC of phomoparagin D (**5**).

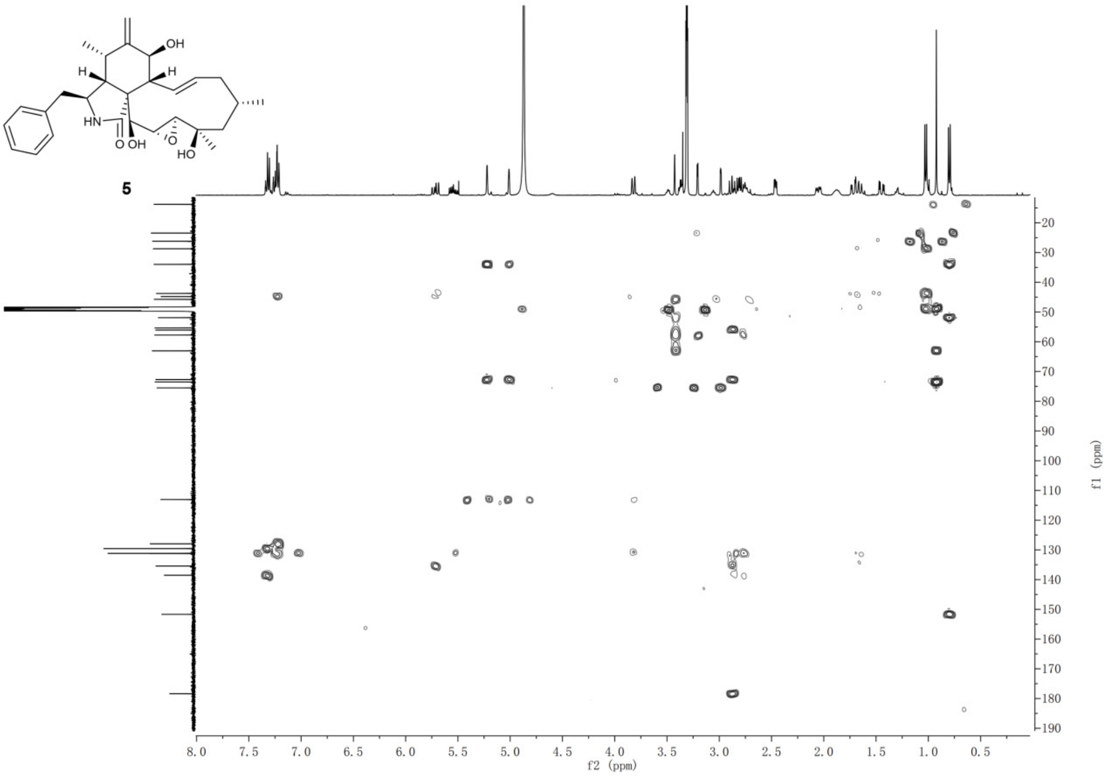

Figure S14. HMBC of phomoparagin D (**5**).

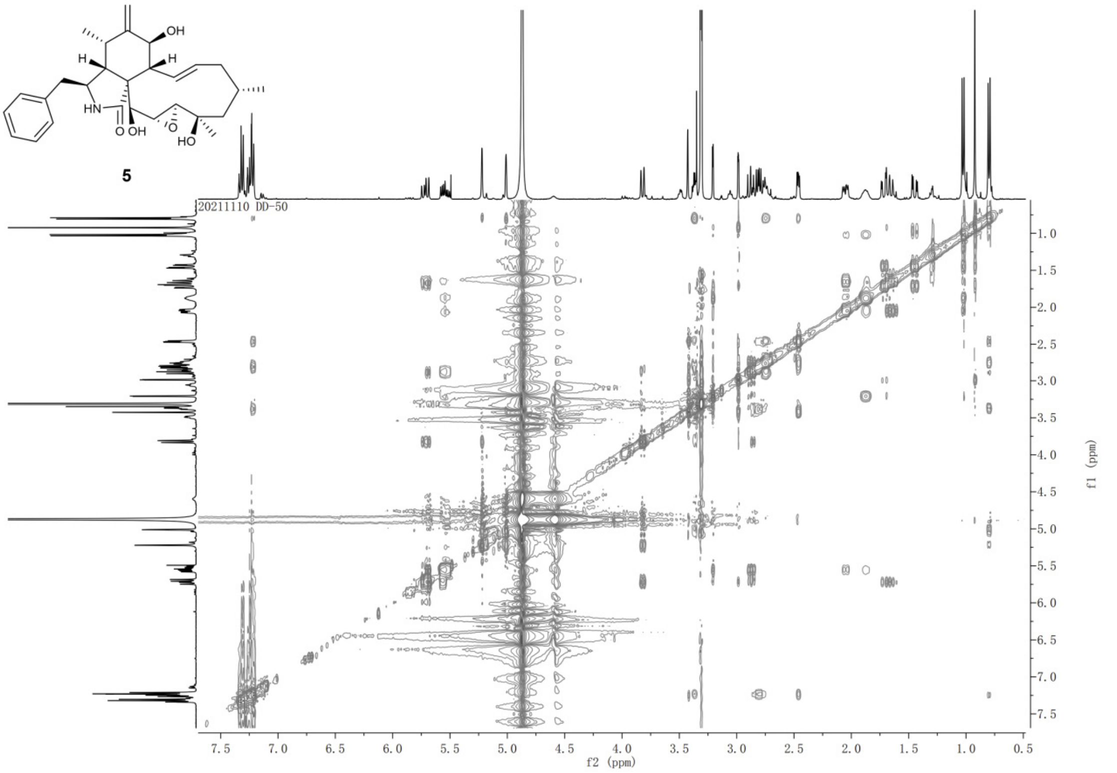

Figure S15. NOSEY of phomoparagin D (**5**).

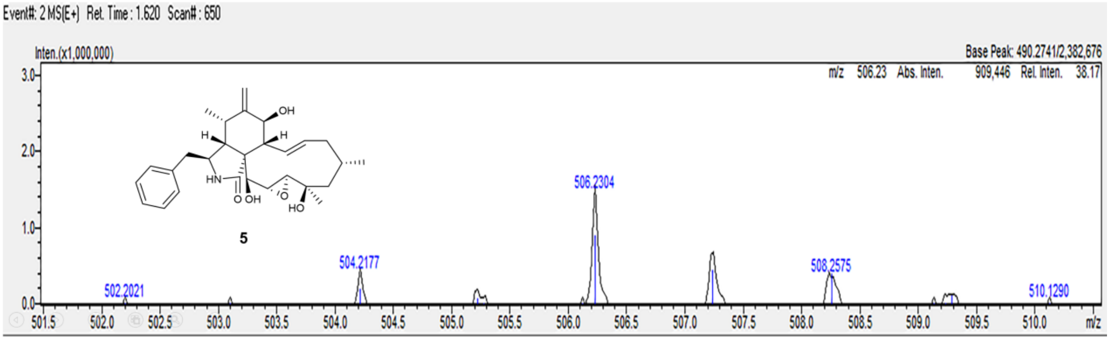

Figure S16. HR-ESI-MS of phomoparagin D (**5**).

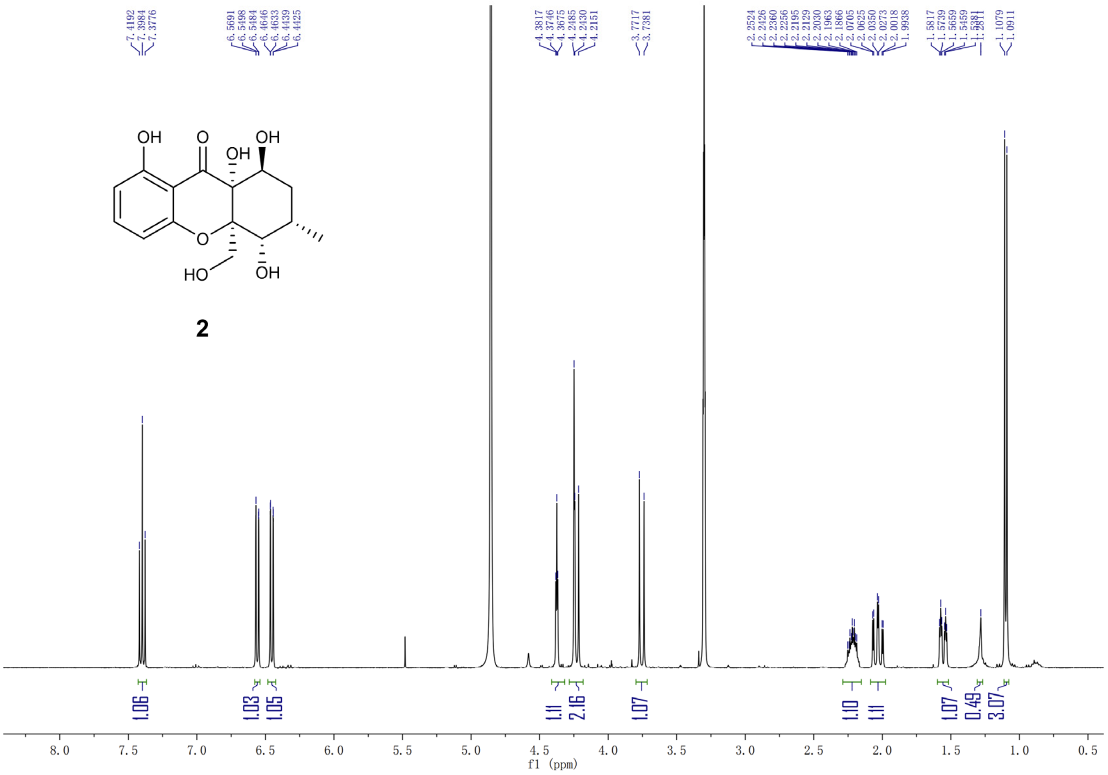

Figure S17. ^{1}H-NMR of phaseolorin D (**2**).

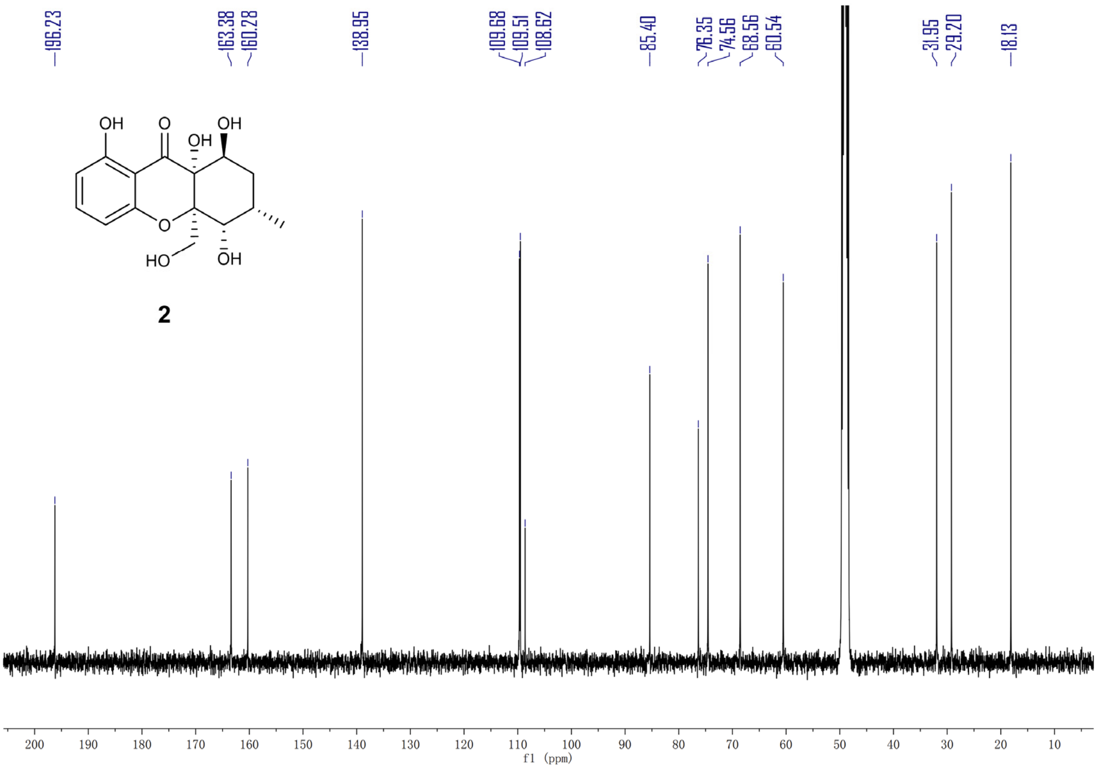

Figure S18. ^{13}C-NMR of phaseolorin D (**2**).

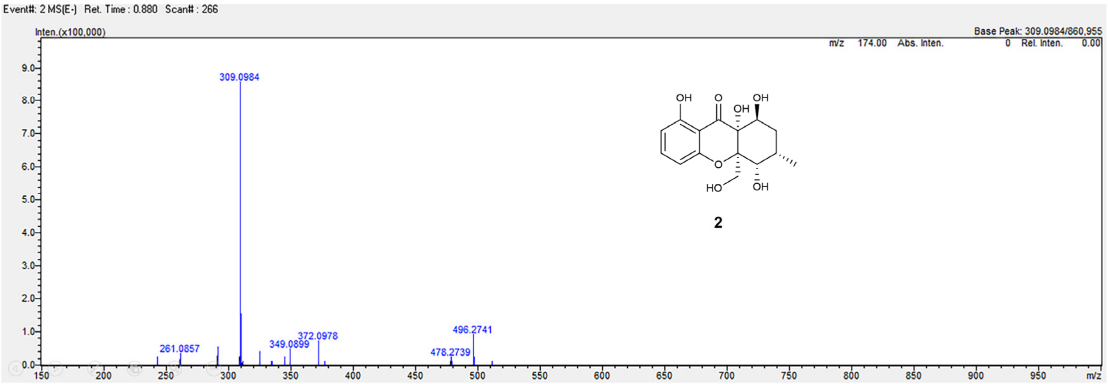

Figure S19. HR-ESI-MS of phaseolorin D (**2**).

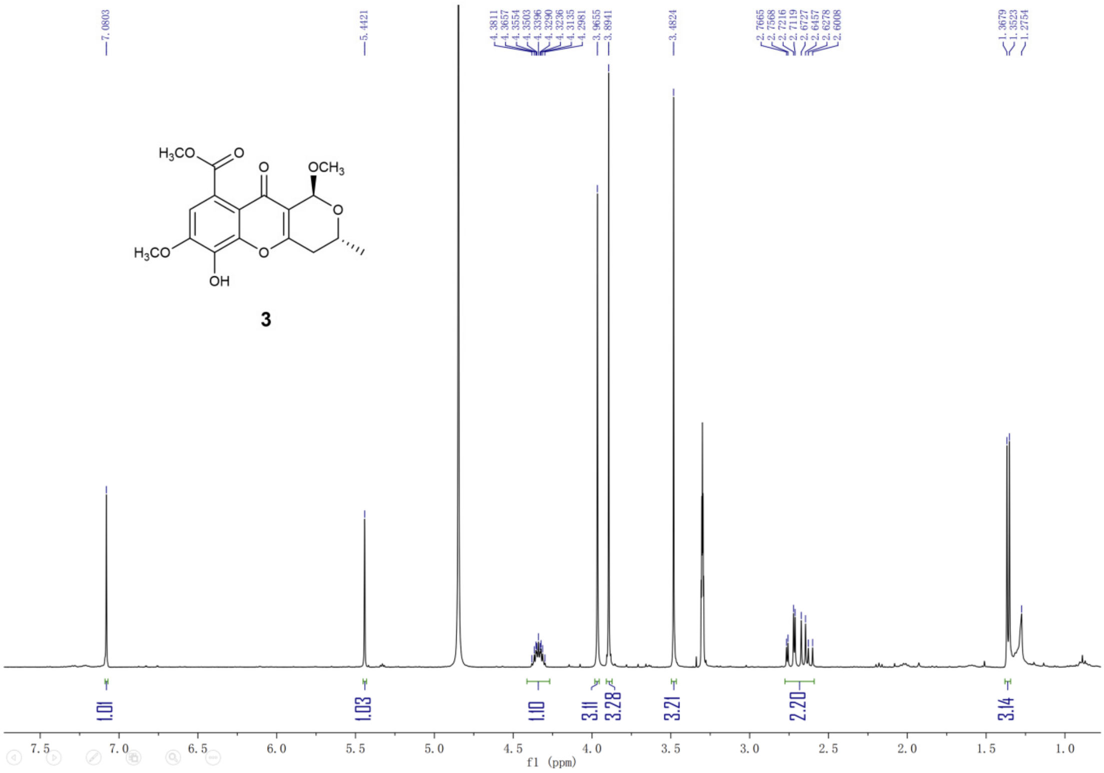

Figure S20. ^{1}H-NMR of chaetochromone B (**3**).

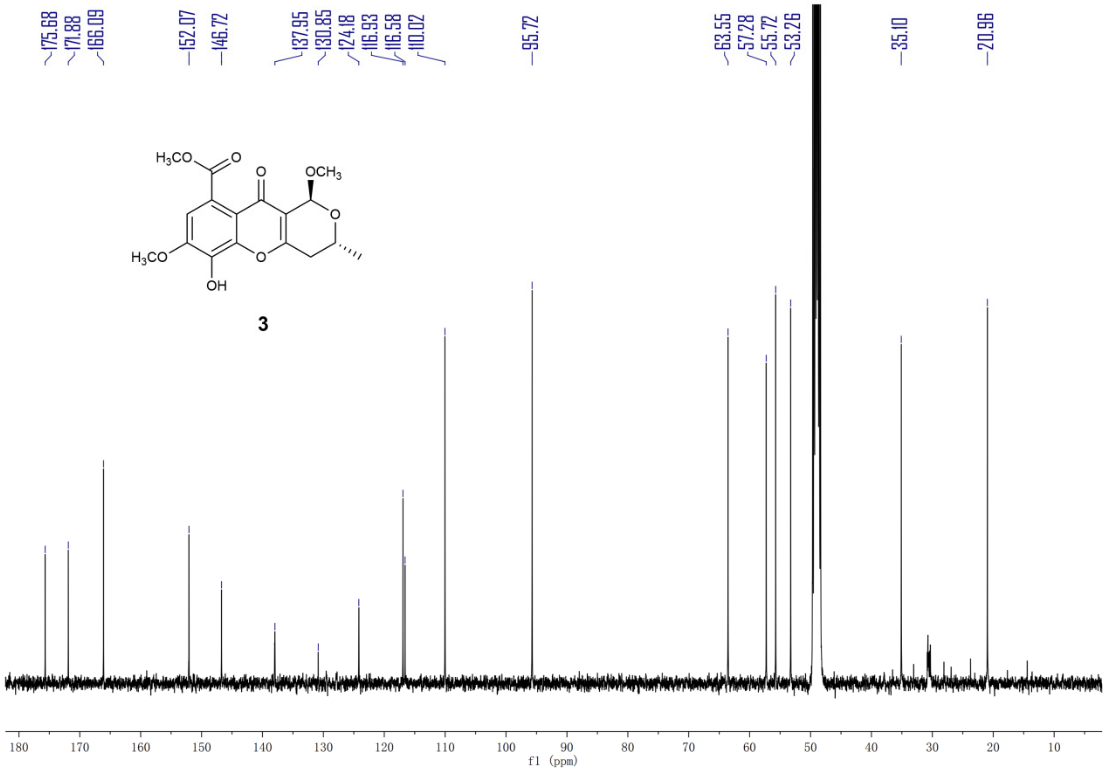

Figure S21. ^{13}C-NMR of chaetochromone B (**3**).

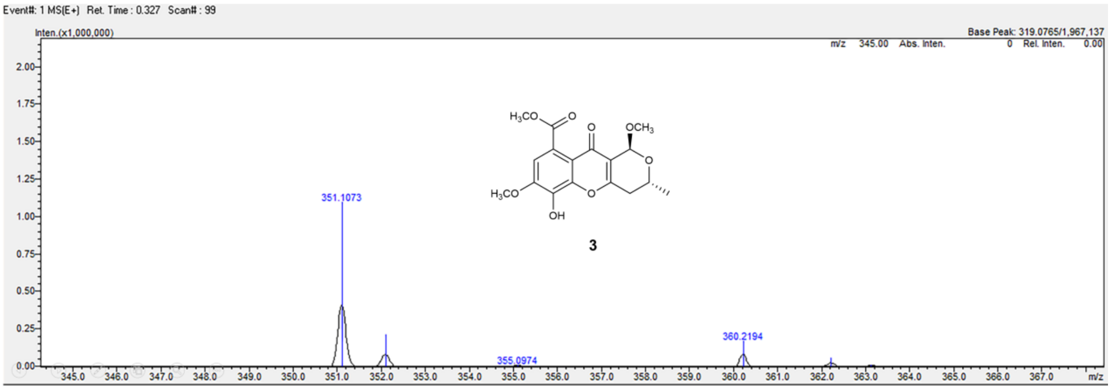

Figure S22. HR-ESI-MS of chaetochromone B (**3**).

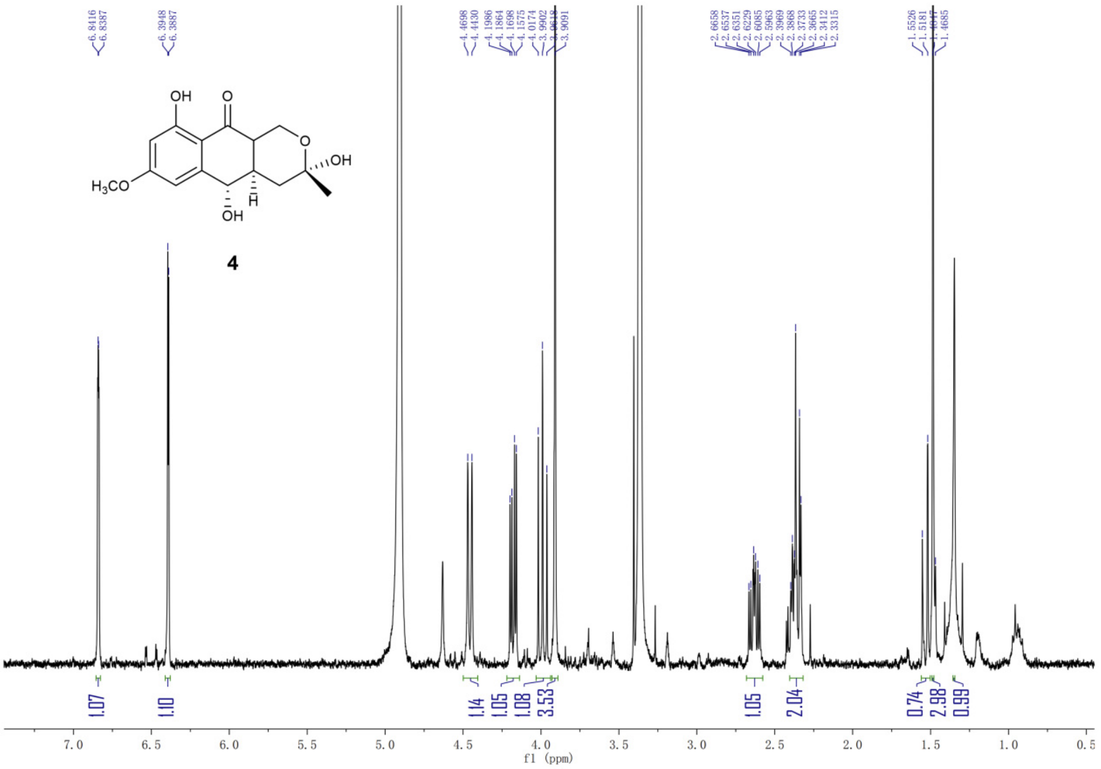

Figure S23. ^{1}H-NMR of pleosporalin D (**4**).

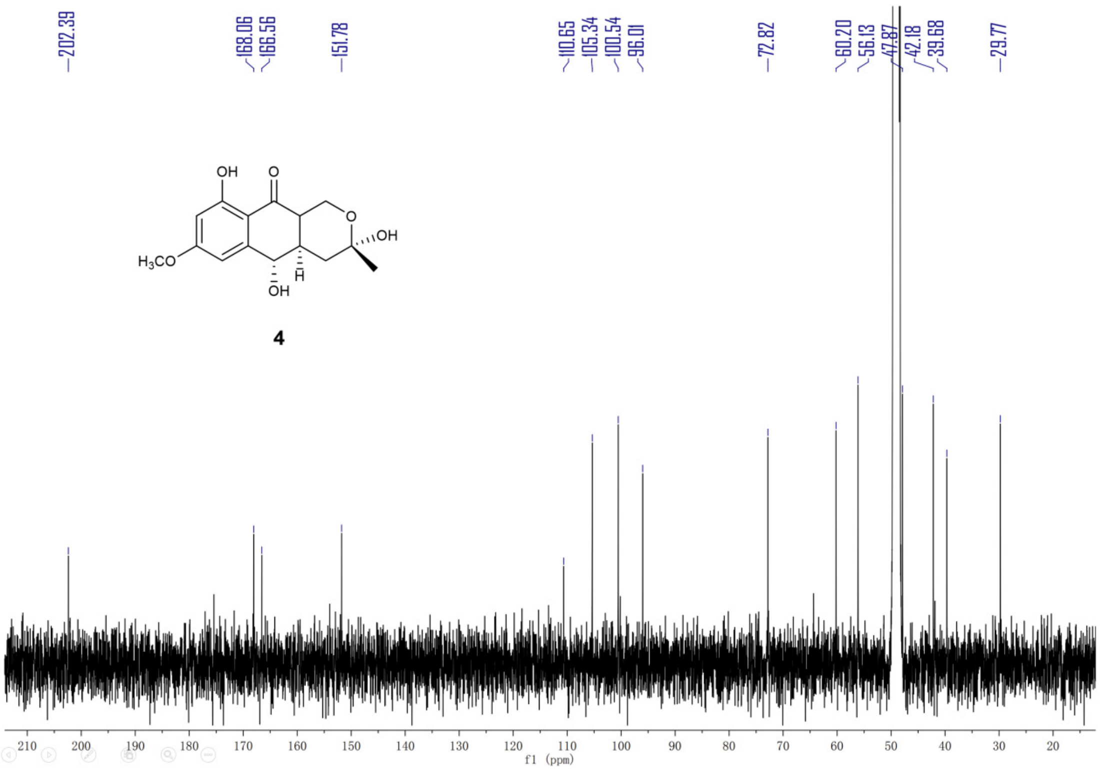

Figure S24. ^{13}C-NMR of pleosporalin D (**4**).

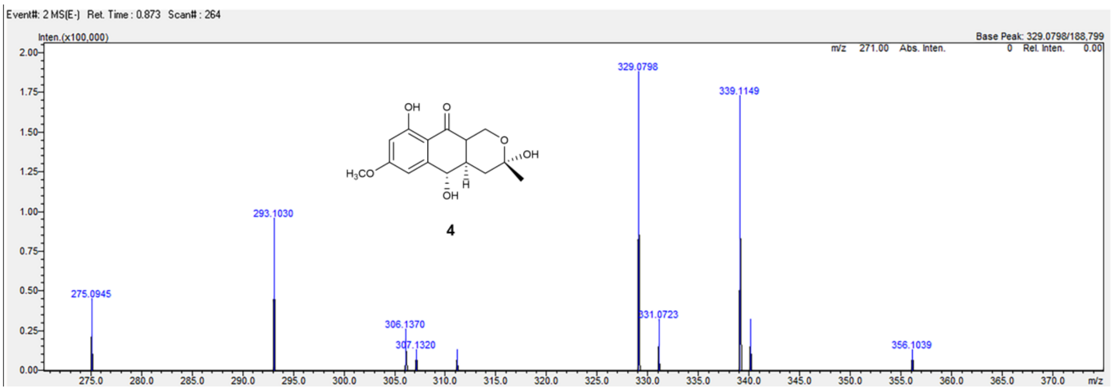

Figure S25. HR-ESI-MS of pleosporalin D (**4**).

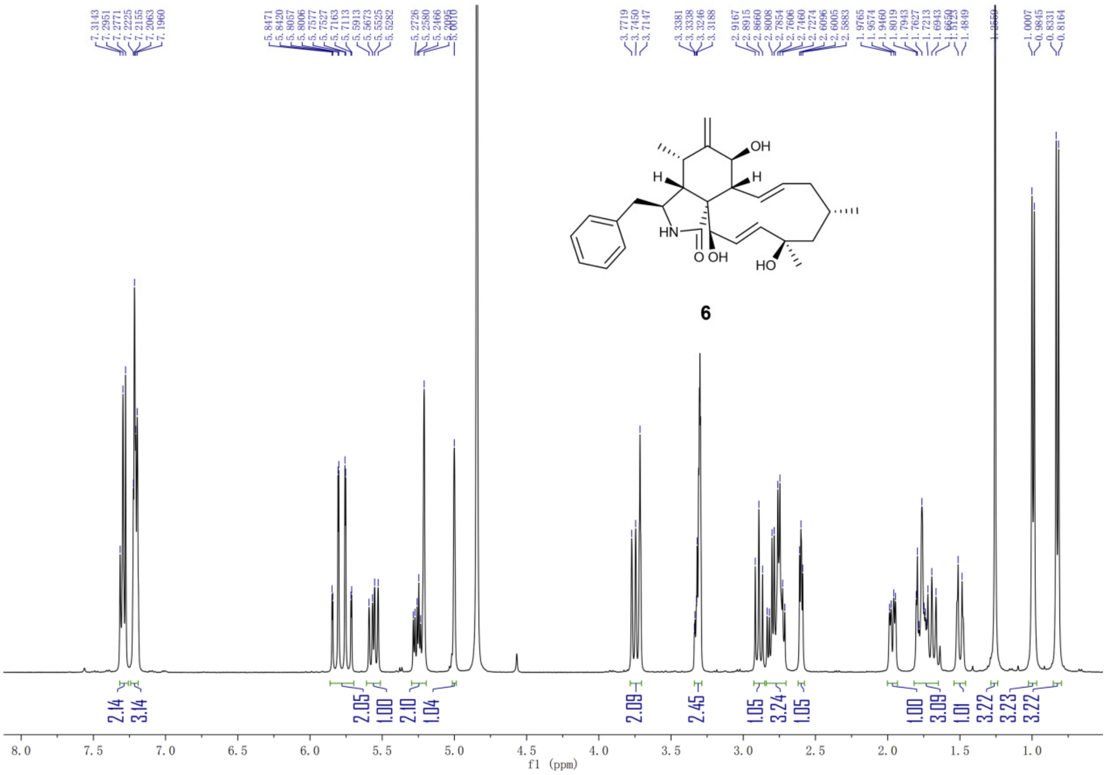

Figure S26. ^{1}H-NMR of cytochalasin J (**6**).

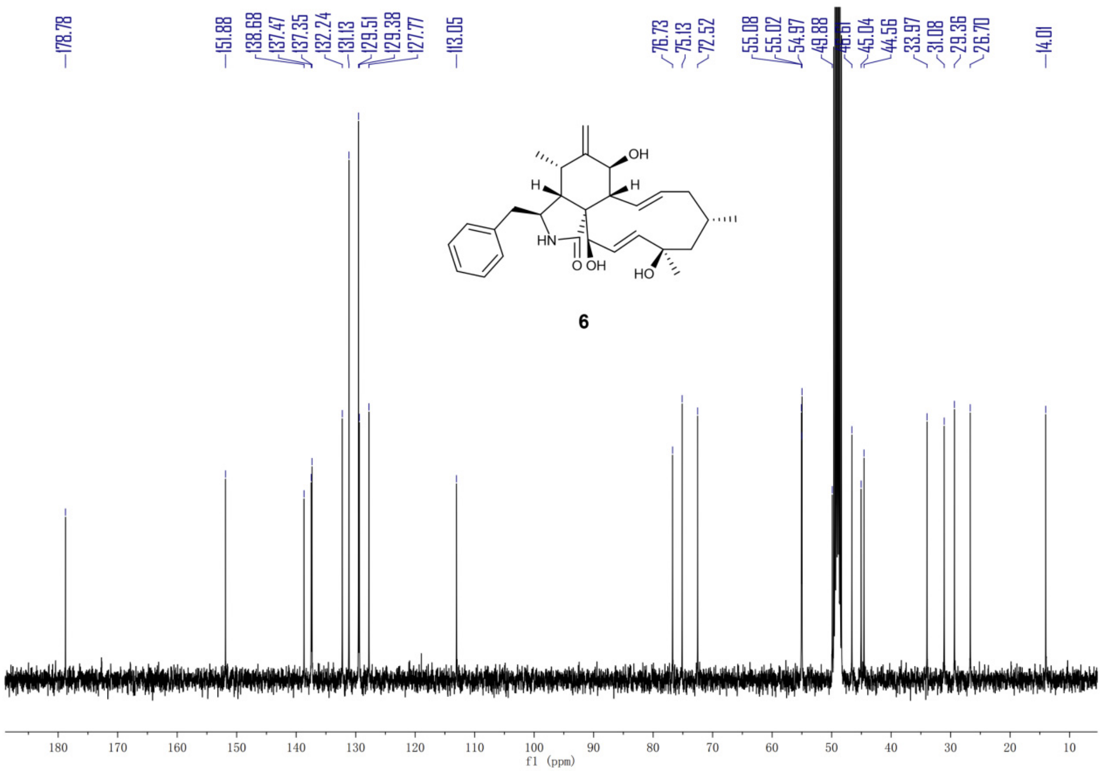

Figure S27. ^{13}C-NMR of cytochalasin J (**6**).

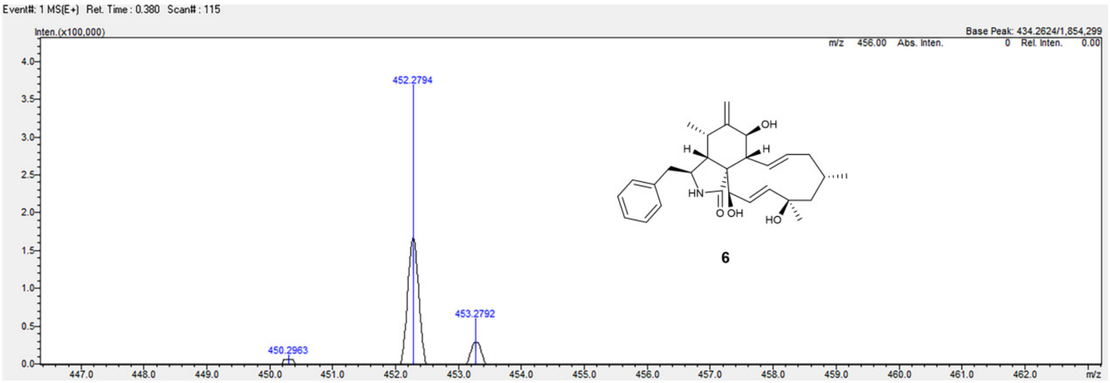

Figure S28. HR-ESI-MS of cytochalasin J (**6**).

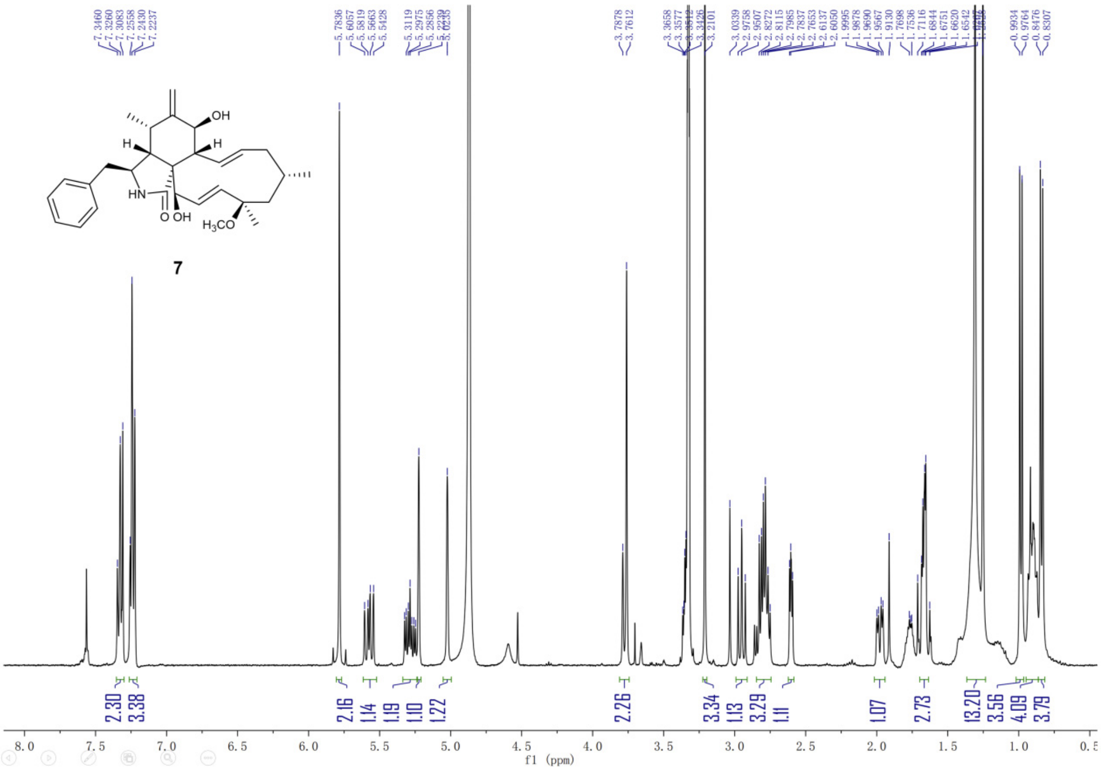

Figure S29. ^{1}H-NMR of cytochalasin J$_1$ (**7**).

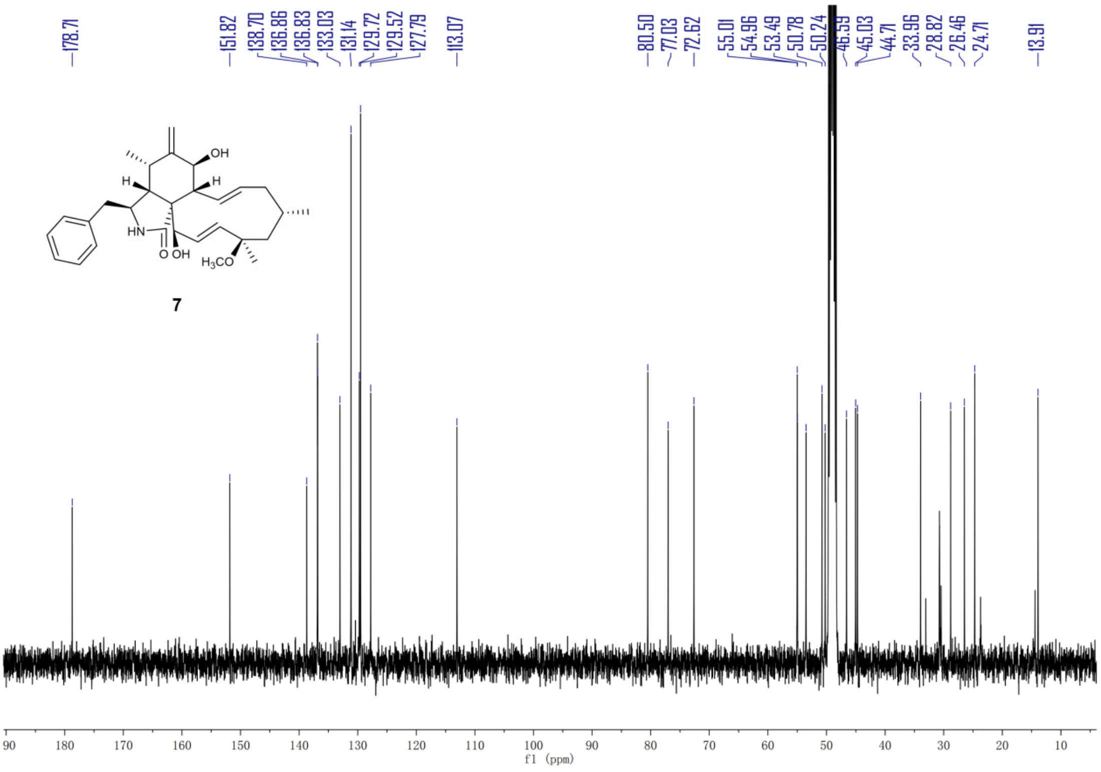

Figure S30. ^{13}C-NMR of cytochalasin J$_1$ (**7**).

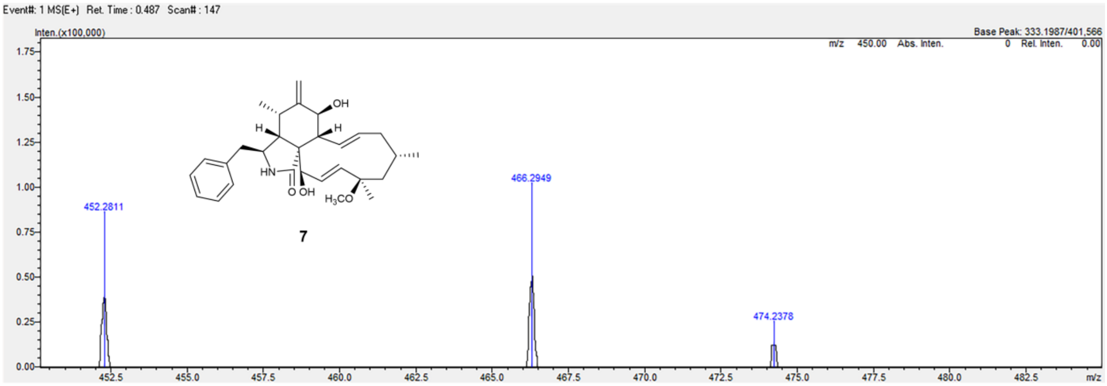

Figure S31. HR-ESI-MS of cytochalasin J$_1$ (**7**).

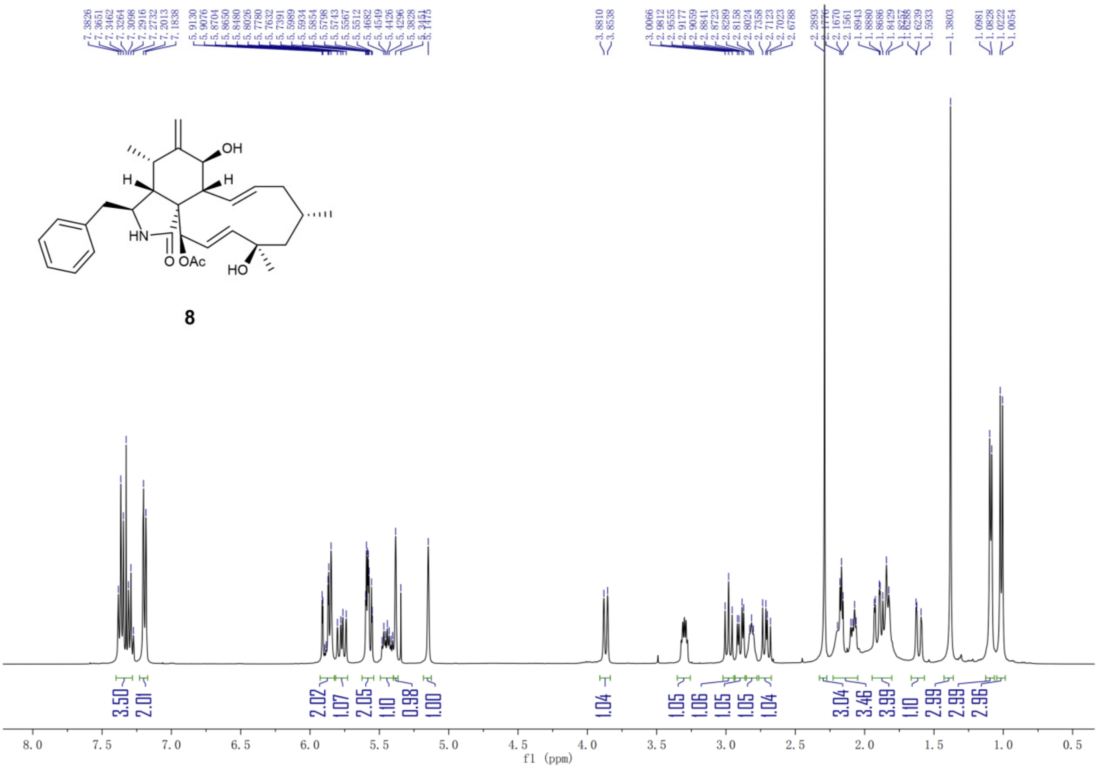

Figure S32. ^{1}H-NMR of cytochalasin H (**8**).

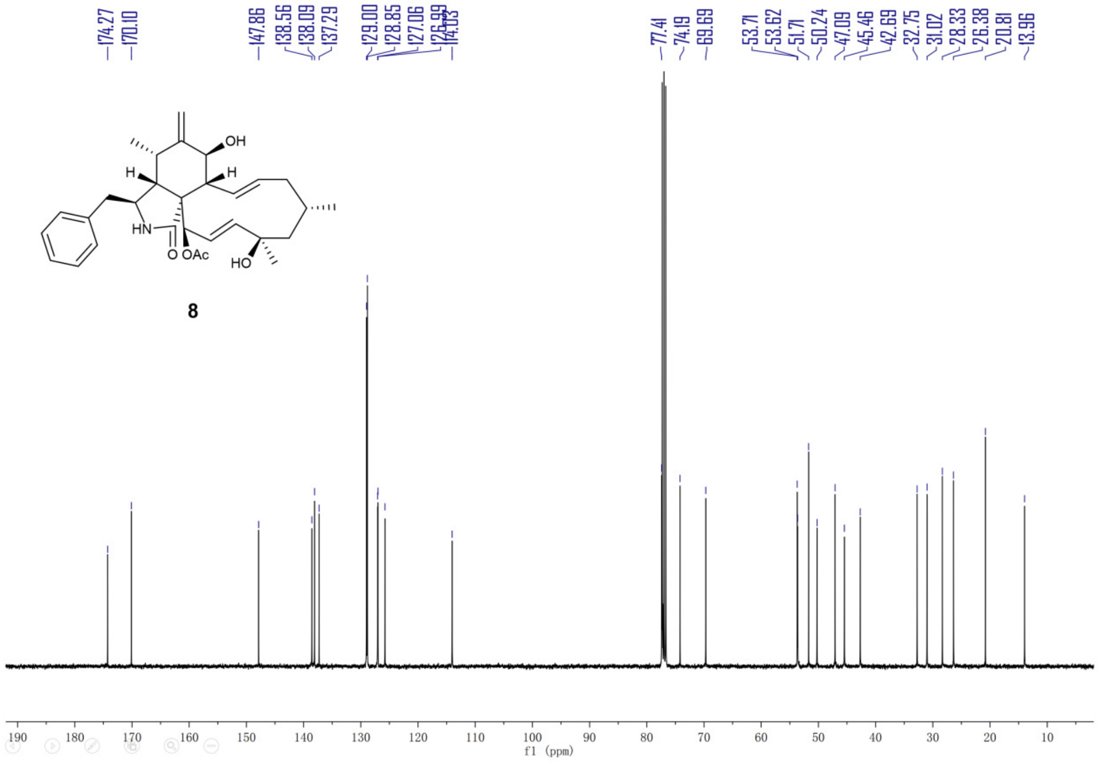

Figure S33. ^{13}C-NMR of cytochalasin H (**8**).

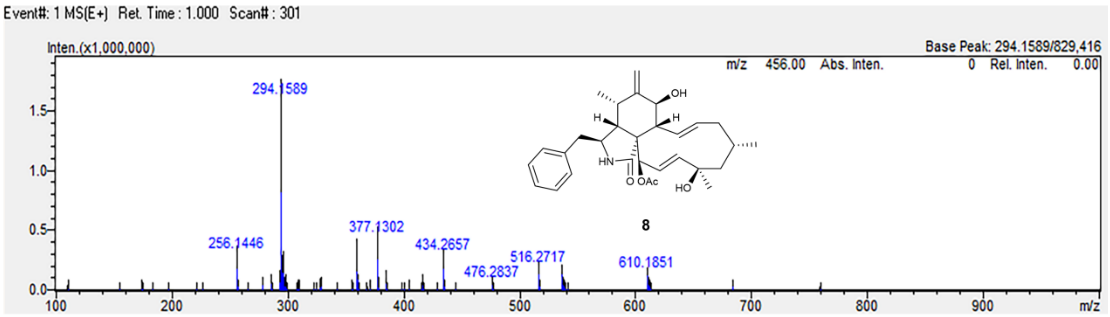

Figure S34. HR-ESI-MS of cytochalasin H (**8**).

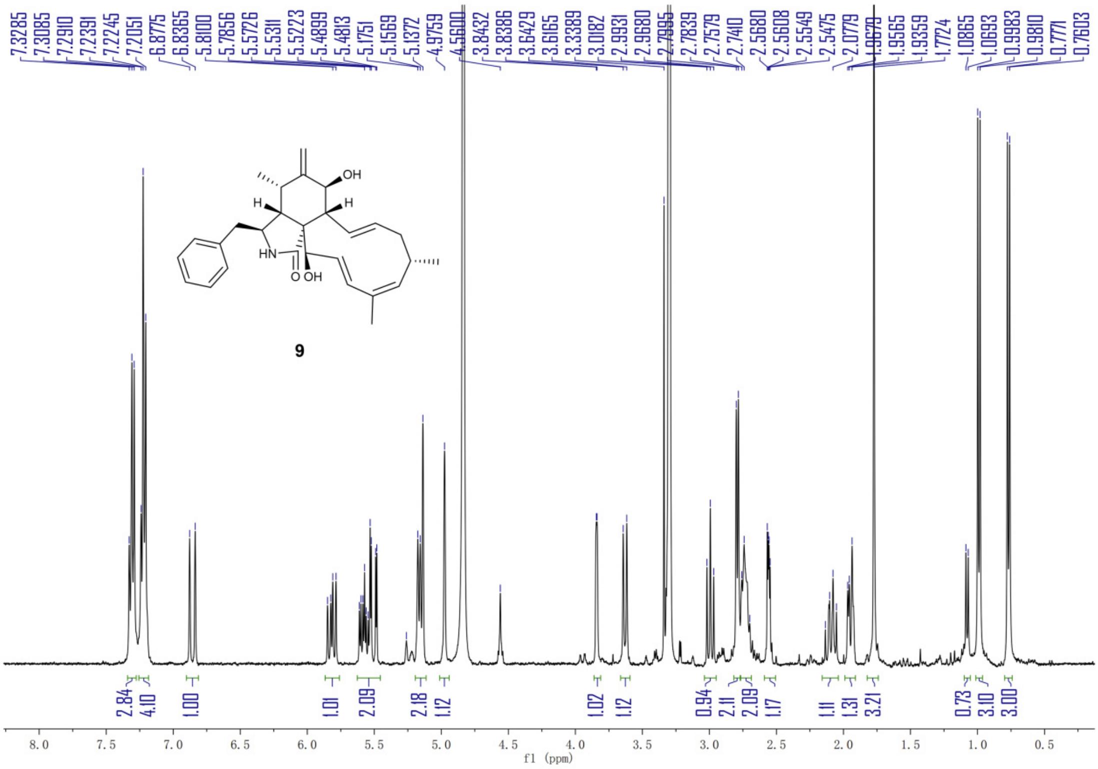

Figure S35. ^{1}H-NMR of cytochalasin J$_2$ (**9**).

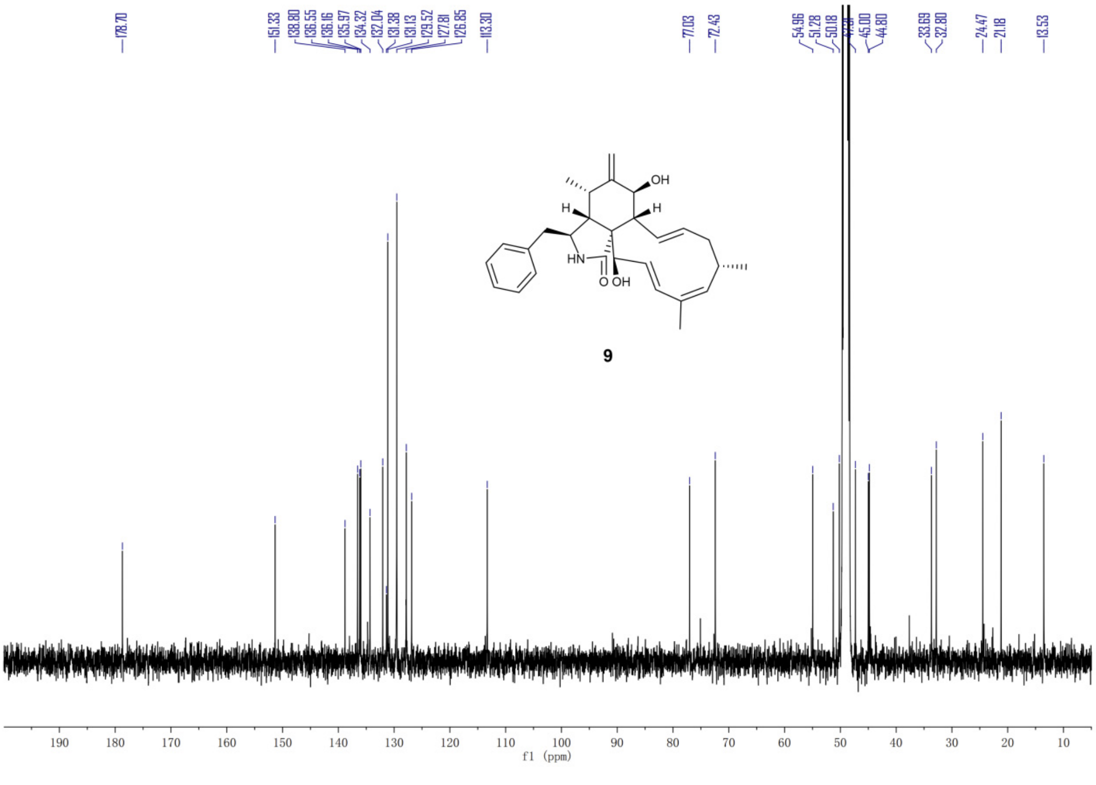

Figure S36. ^{13}C-NMR of cytochalasin J$_2$ (**9**).

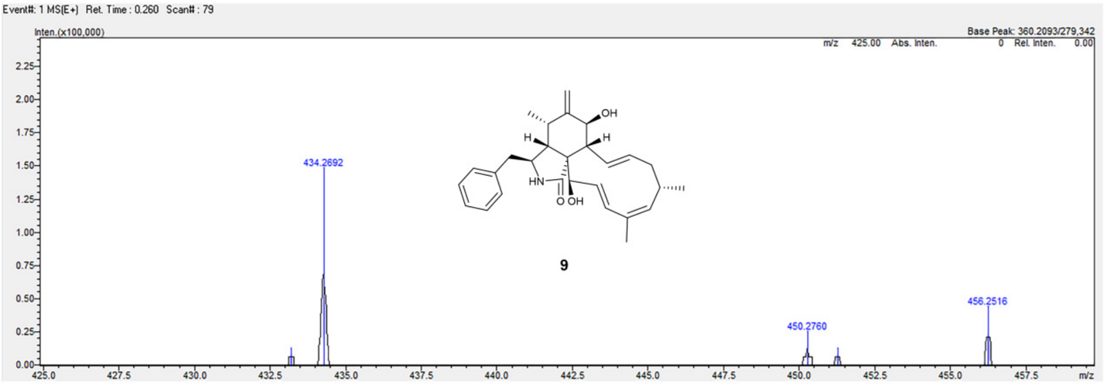

Figure S37. HR-ESI-MS of cytochalasin J$_2$ (**9**).

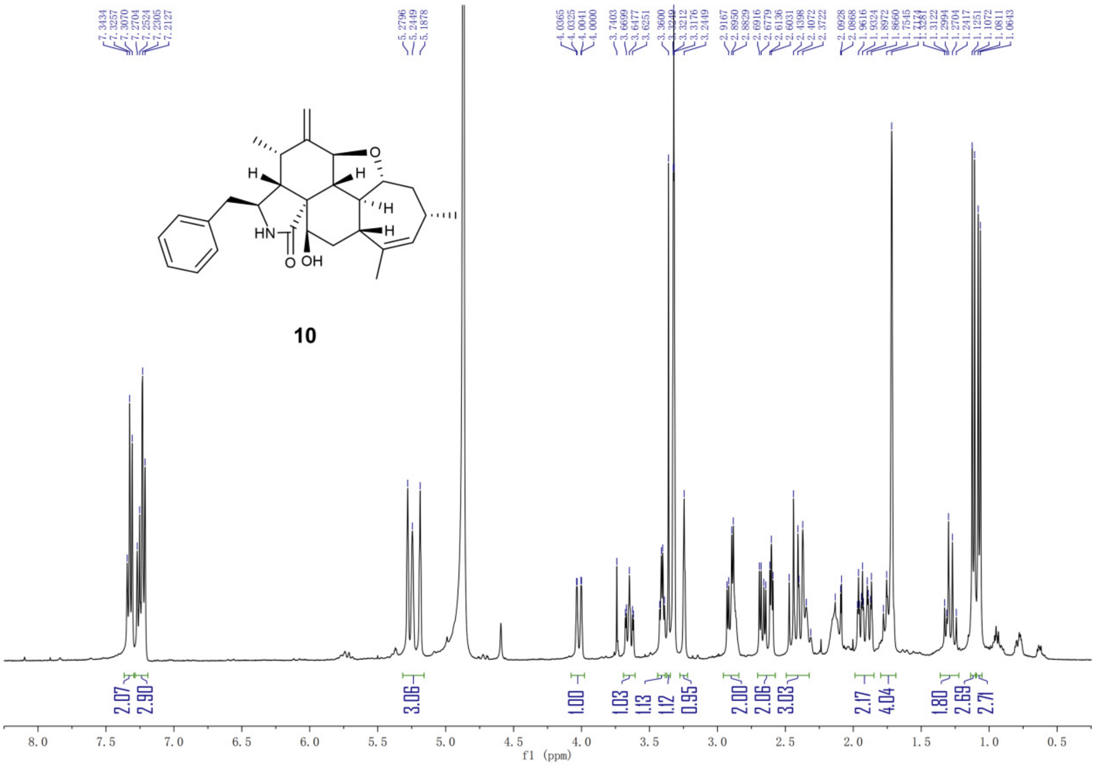

Figure S38. ^{1}H-NMR of cytochalasin J$_3$ (**10**).

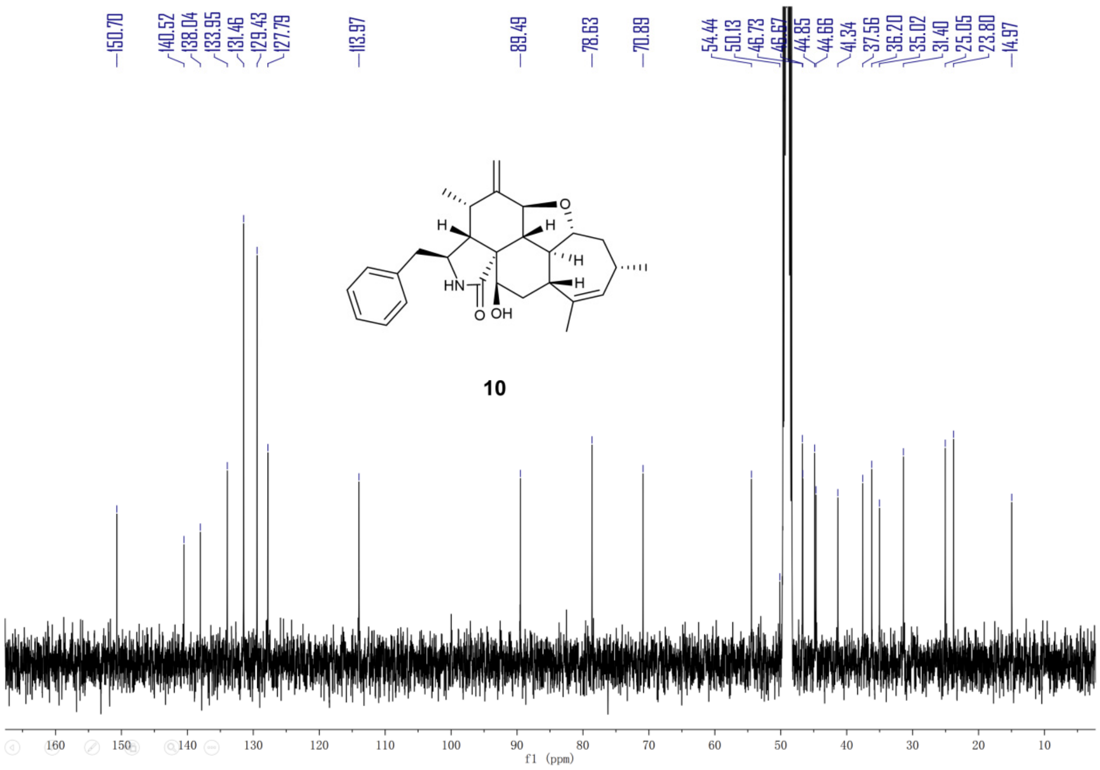

Figure S39. ^{13}C-NMR of cytochalasin J$_3$ (**10**).

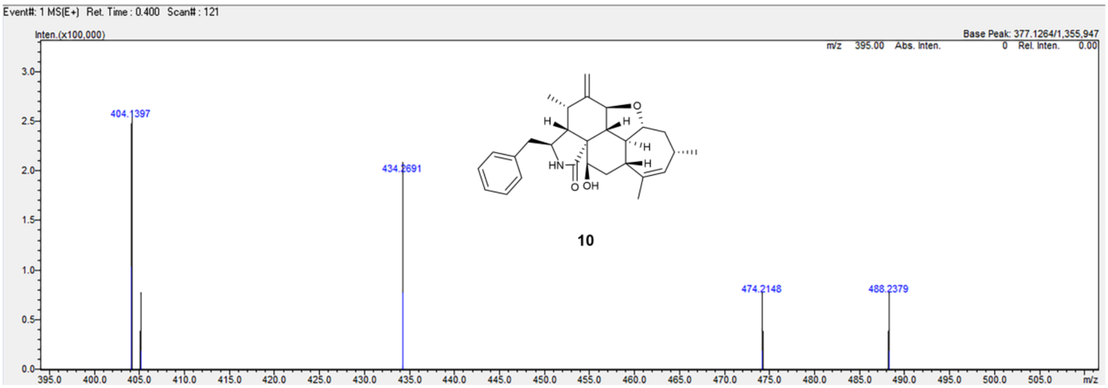

Figure S40. HR-ESI-MS of cytochalasin J$_3$ (**10**).

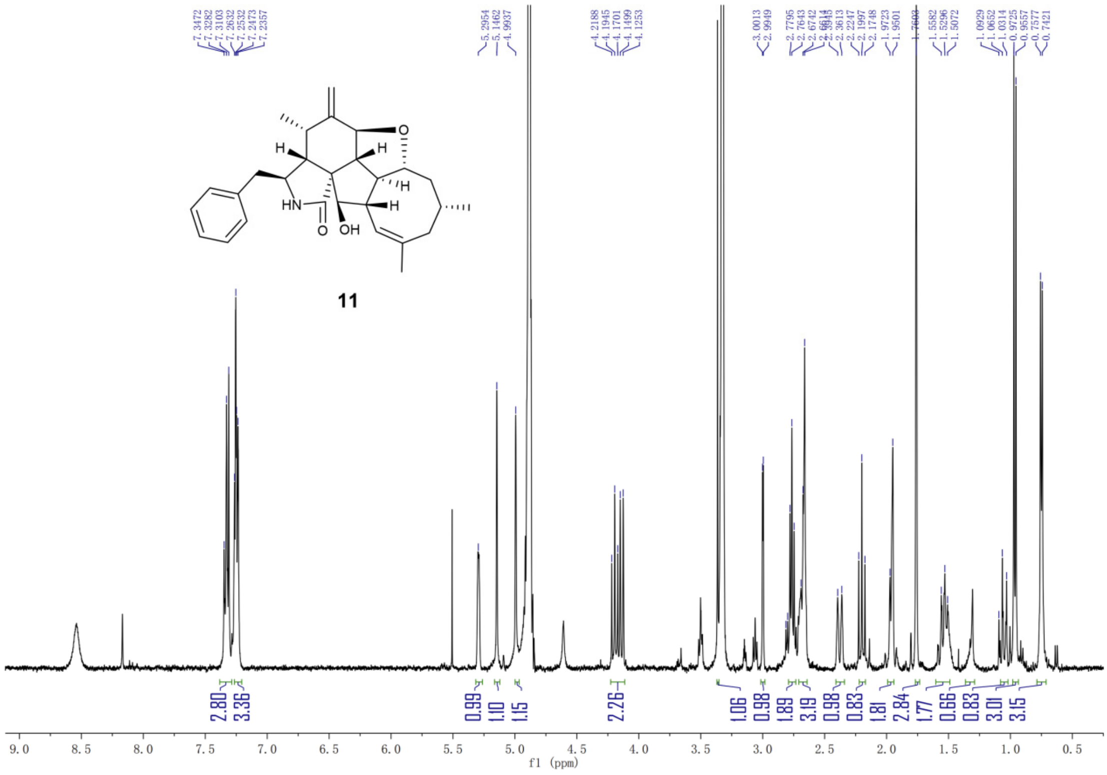

Figure S41. ^{1}H-NMR of phomopchalasin D (**11**).

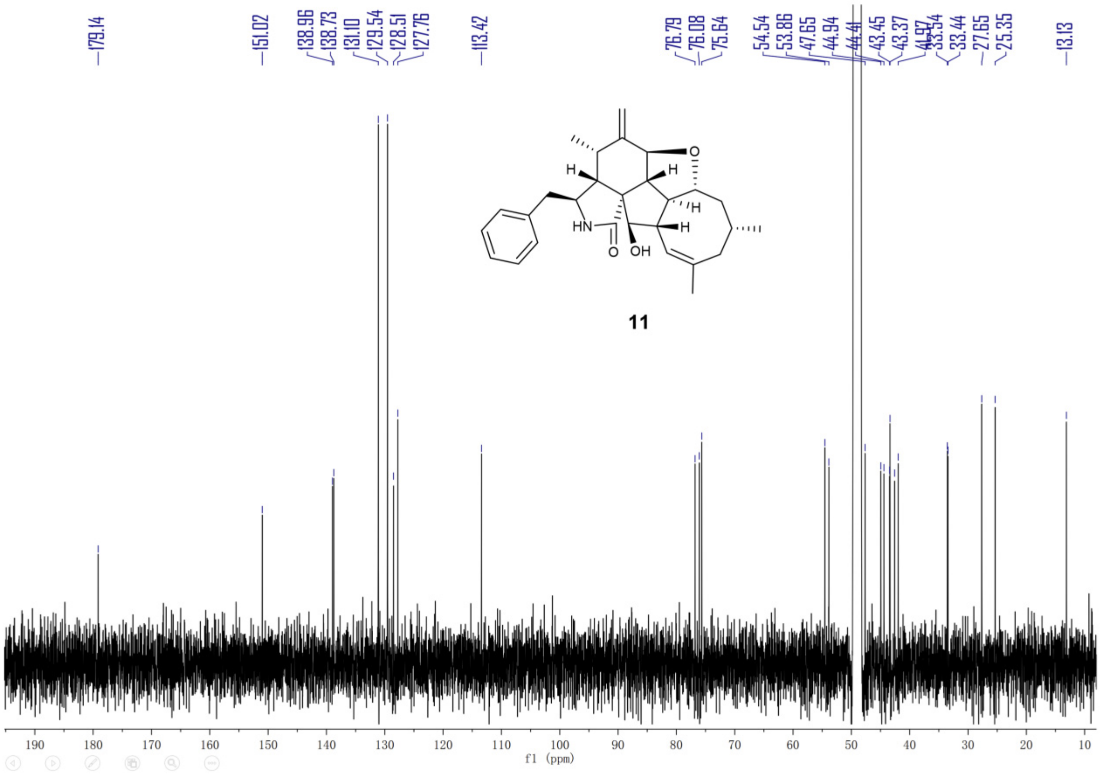

Figure S42. ^{13}C-NMR of phomopchalasin D (**11**).

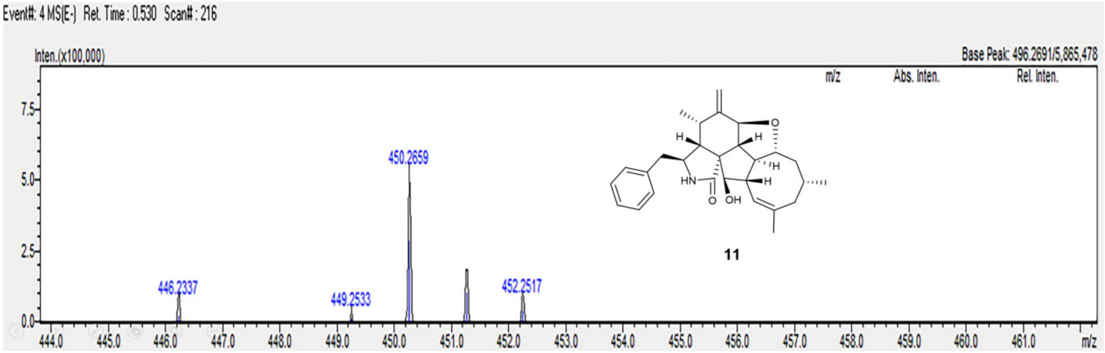

Figure S43. HR-ESI-MS of phomopchalasin D (**11**).

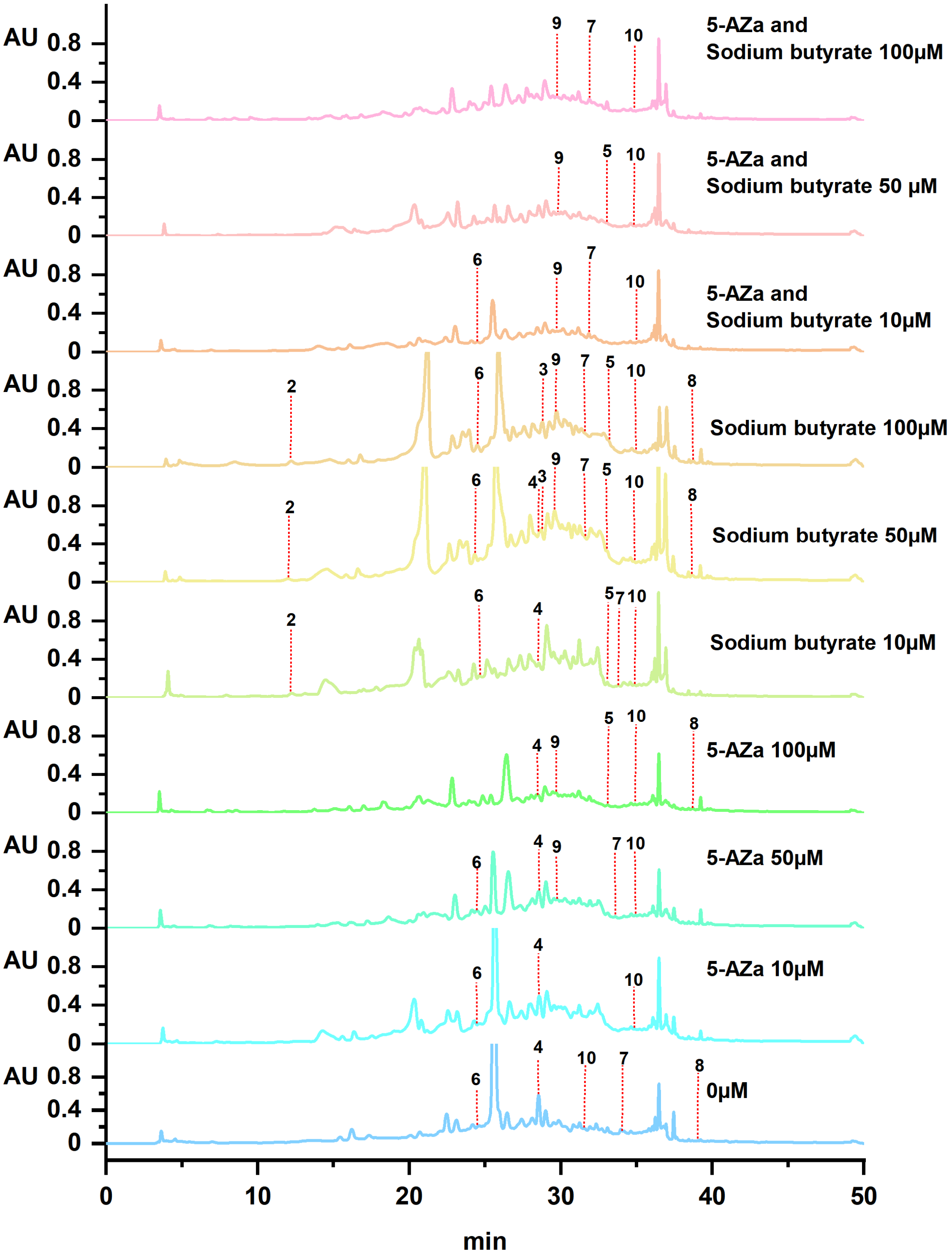

Figure S44. Overlay of HPLC profiles of EtOAc extracts of *Phomopsis asparagi* DHS-48 cultivated in PDA treated with different epigenetic agents.

Figure S45. HPLC spectrum for the purity of tested compounds**.** HPLC chromatograms: C18 column (Agilent Technologies 10 mm×250 mm). Solvents: A, H_2O; B, MeOH. Linear gradient: 0 min,60% B; 40 min, 100% B. Temperature 25°C. Flow rate 2 mL/min. UV detection

at λ = 210 nm.

Compound 1

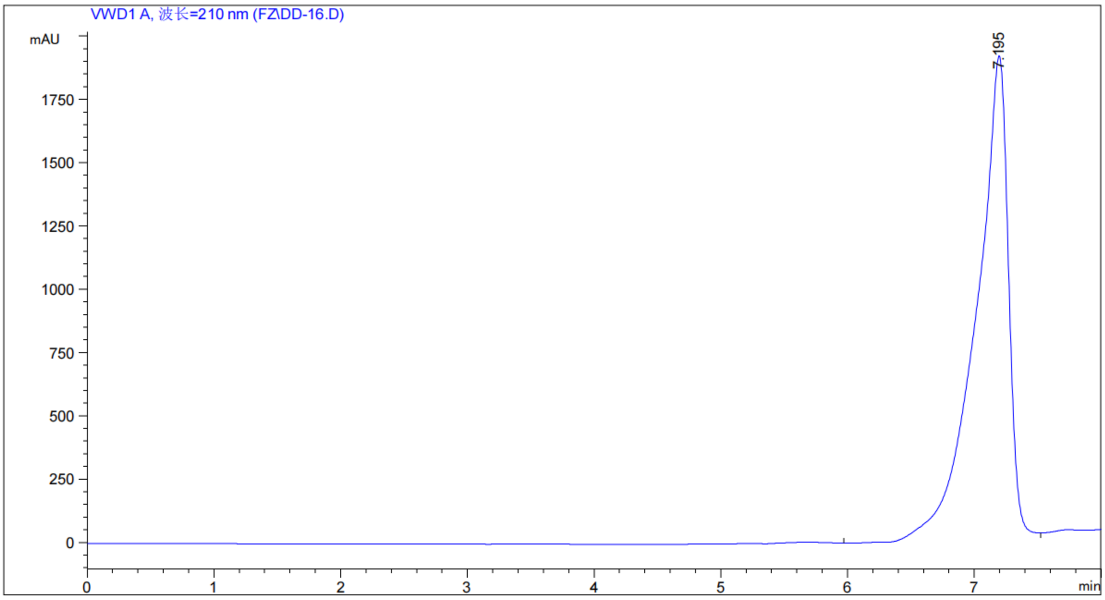

Peak Retention time	Peak type	Peak width	Peak area	Peak height	Peak area%
# [min]		[min]	mAU *s	[mAU]	%
1 7.195	VV	0.2547	3.52018e4	1920.09363	100.0000
sum :			3.52018e4	1920.09363	

Compound 2

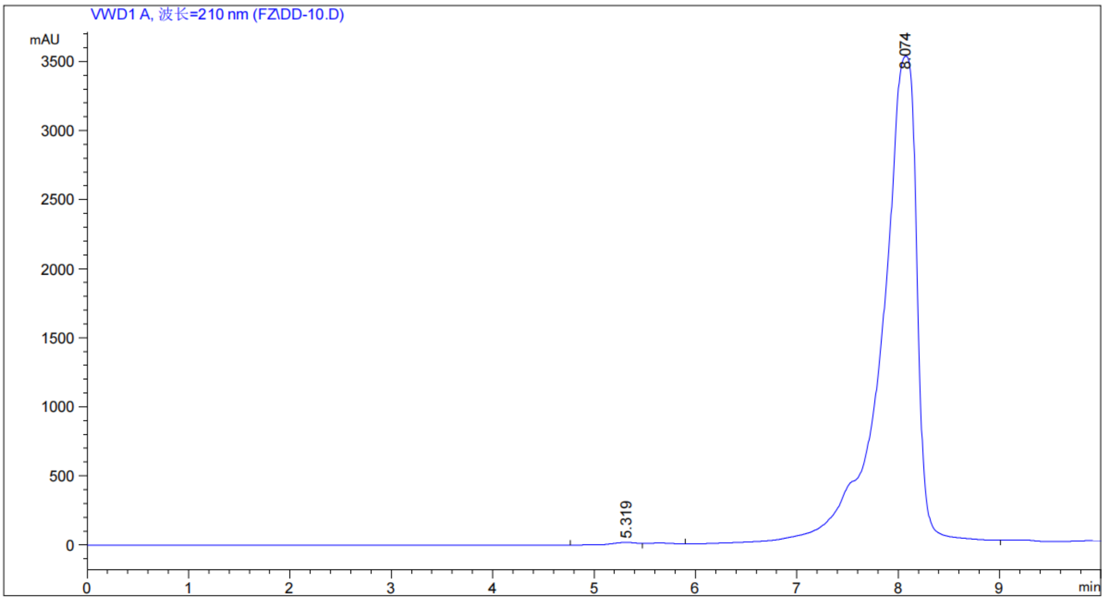

Peak Retention time Peak type Peak width Peak area Peak height Peak area%

#	[min]		[min]	mAU *s	[mAU]	%
1	7.195	VV	0.2547	3.52018e4	1920.09363	100.0000
sum :				3.52018e4	1920.09363	

Compound 3

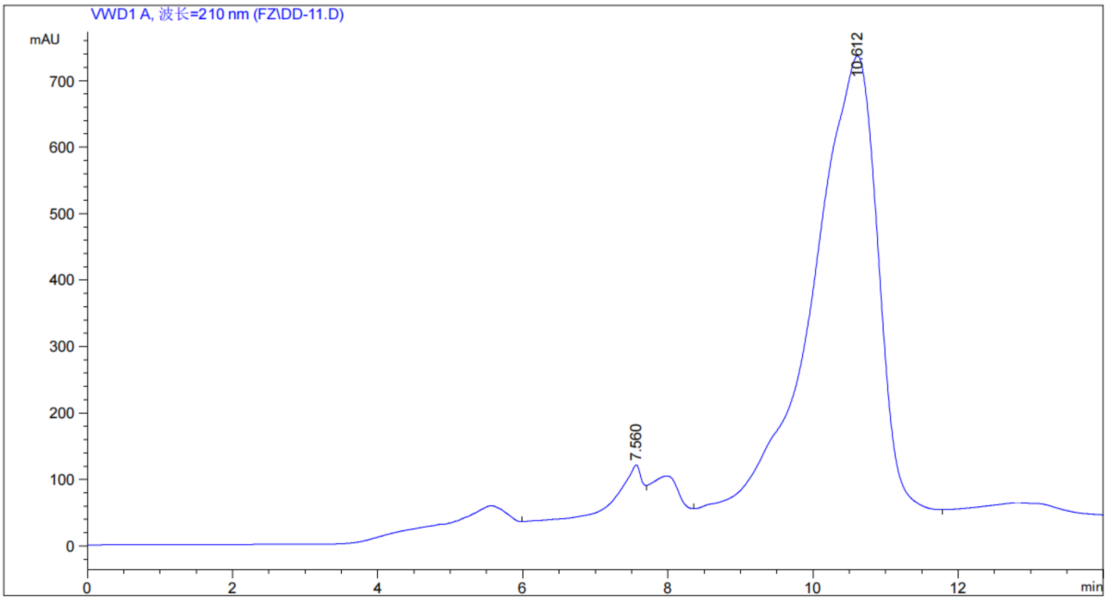

Peak	Retention time	Peak type	Peak width	Peak area	Peak height	Peak area%
#	[min]		[min]	mAU *s	[mAU]	%
1	7.560	VV	0.5985	5753.92334	119.12096	9.8707
2	10.612	VV	0.9628	5.25392e4	734.33246	90.1293
sum :				5.82932e4	853.45342	

Compound 4

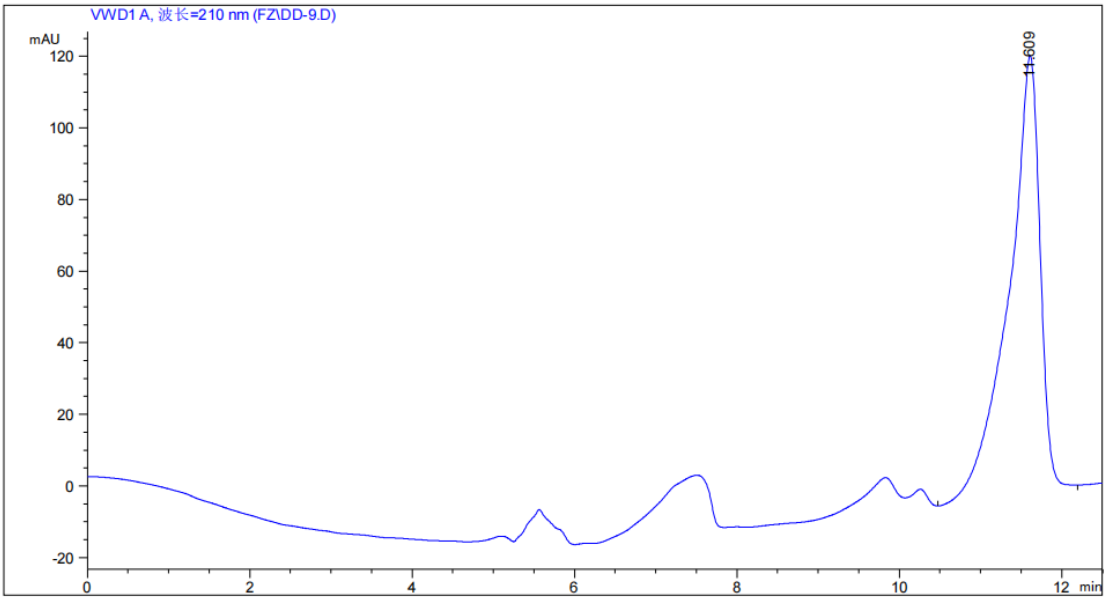

Peak	Retention time	Peak type	Peak width	Peak area	Peak height	Peak area%
#	[min]		[min]	mAU *s	[mAU]	%
1	11.609	VV	0.4622	4645.15332	136.13113	100.0000
sum：				4645.15332	136.13113	

Compound 5

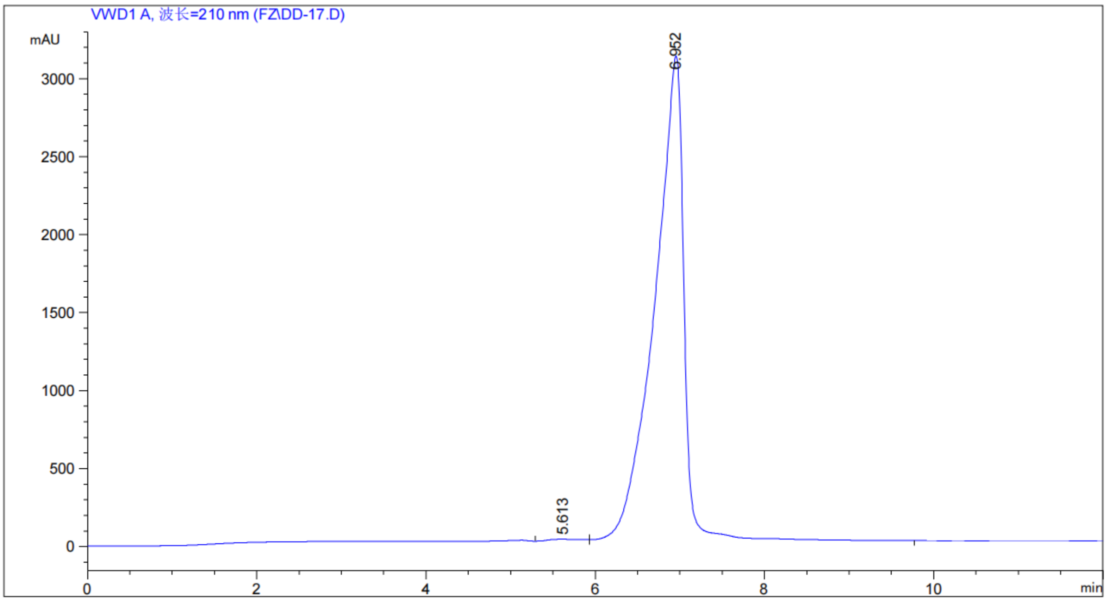

Peak Retention time Peak type Peak width Peak area Peak height Peak area%

#	[min]		[min]	mAU *s	[mAU]	%
1	5.613	VV	0.4863	1497.32666	42.54254	1.7895
2	6.952	VB	0.3521	8.21750e4	3137.66675	98.2105

sum : 8.36724e4 3180.20929

Compound 6

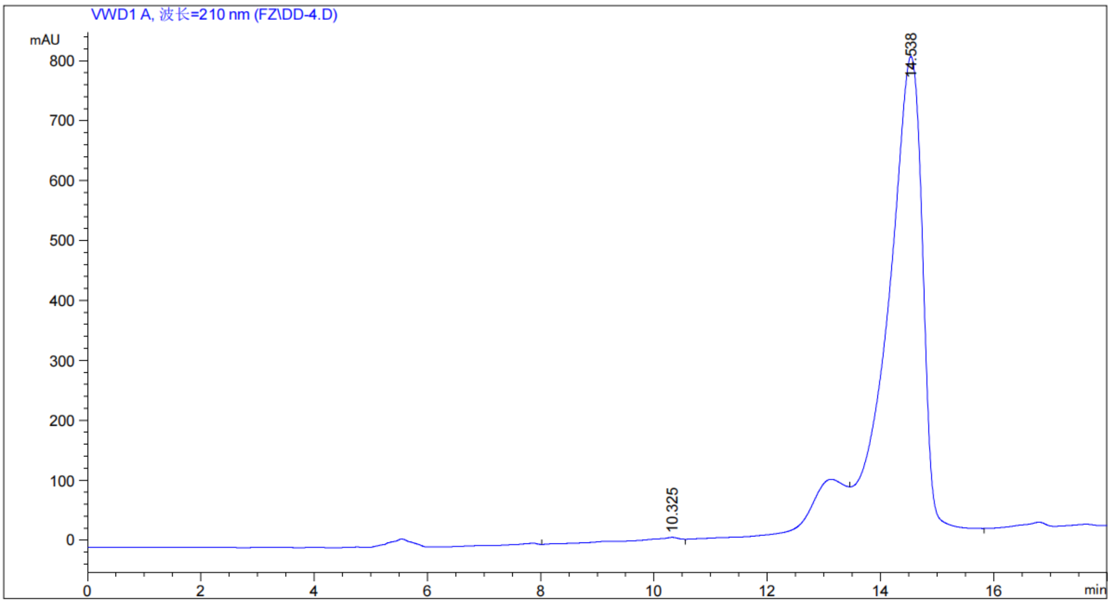

Peak	Retention time	Peak type	Peak width	Peak area	Peak height	Peak area%
#	[min]		[min]	mAU *s	[mAU]	%
1	10.325	VV	1.1577	1638.47131	17.20361	4.2330
2	14.538	VB	0.6616	3.70686e4	819.60638	95.7670

sum : 3.87071e4 836.80999

Compound 7

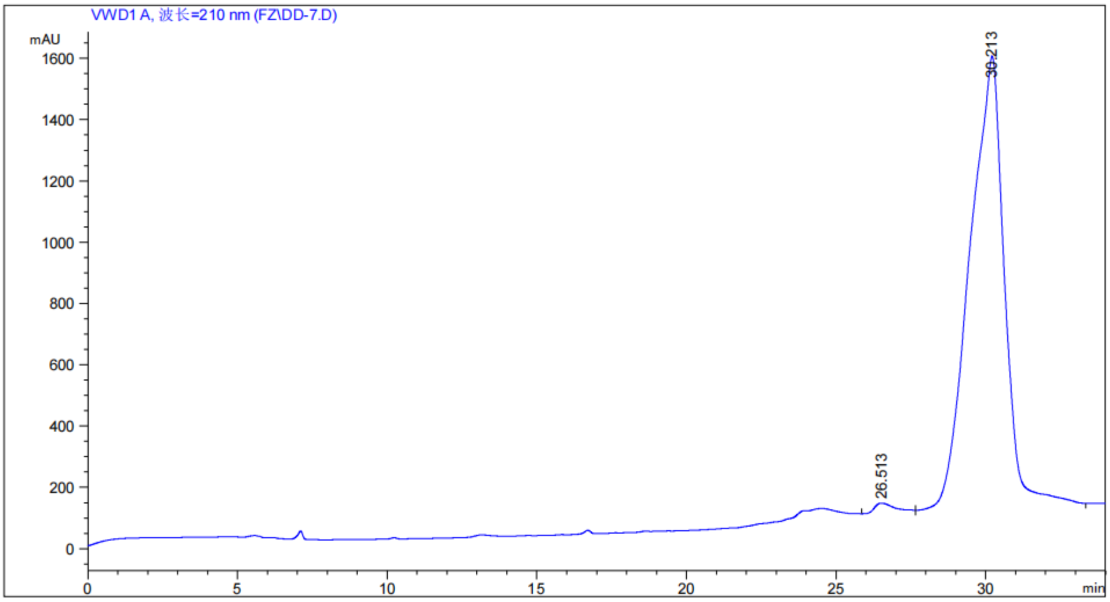

Peak #	Retention time [min]	Peak type	Peak width [min]	Peak area mAU *s	Peak height [mAU]	Peak area% %
1	26.513	VV	0.9434	3352.33447	49.74641	2.6536
2	30.213	VV	1.0923	1.22977e5	1488.04834	97.3464

sum: 1.26330e5 1537.79475

Compound 8

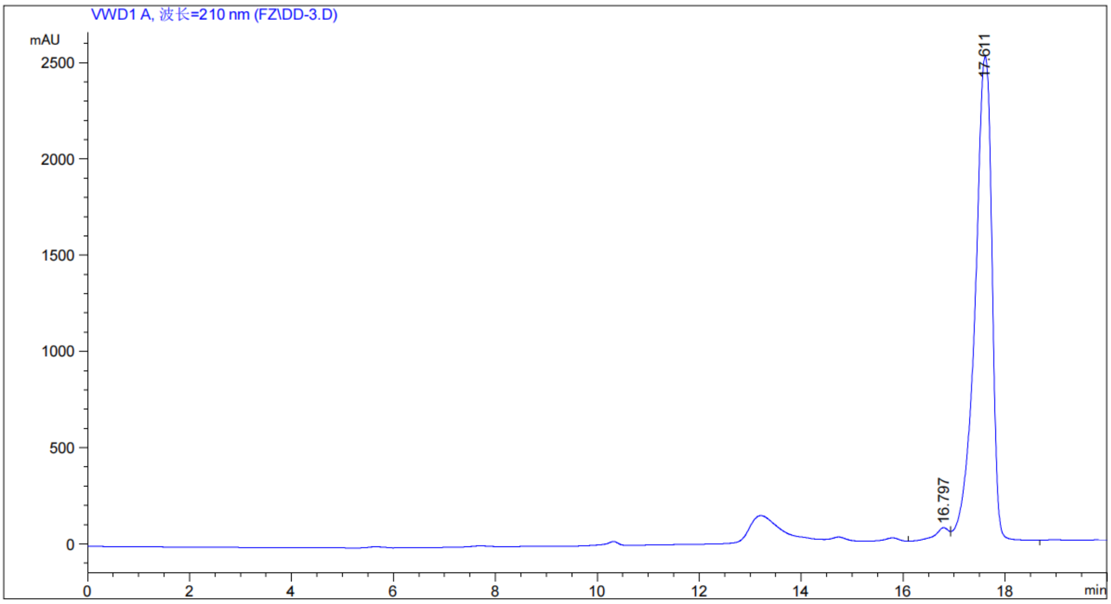

Peak Retention time Peak type Peak width Peak area Peak height Peak area%

#	[min]		[min]	mAU *s	[mAU]	%
1	16.797	VV	0.3978	3117.22534	105.28576	4.7910
2	17.611	VB	0.3647	6.19464e4	2552.15552	95.2090

sum : 6.50637e4 2657.44128

Compound 9

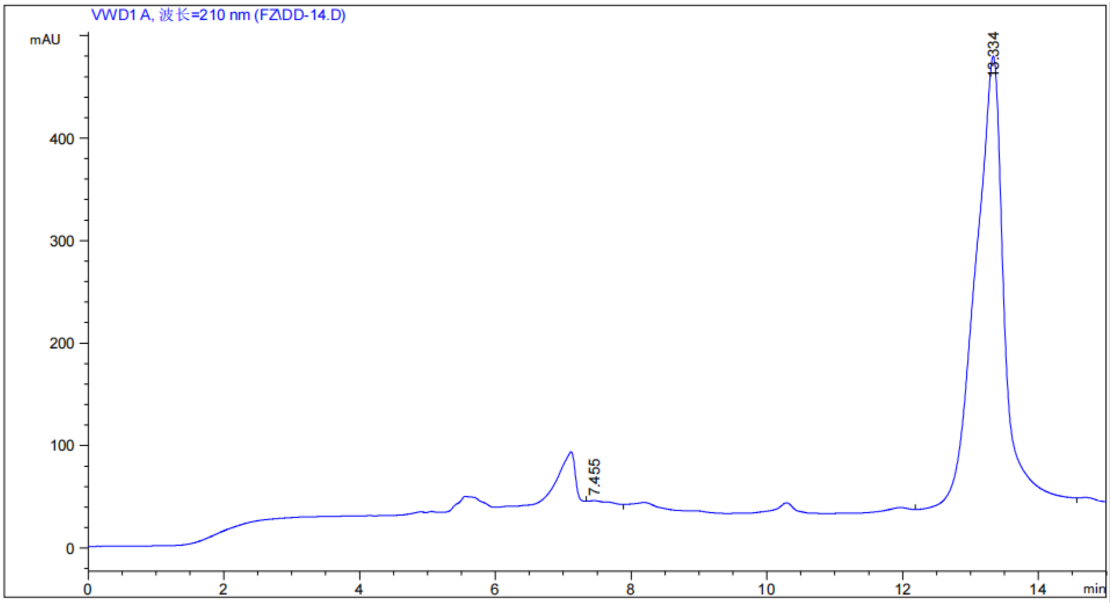

Peak	Retention time	Peak type	Peak width	Peak area	Peak height	Peak area%
#	[min]		[min]	mAU *s	[mAU]	%
1	7.455	VV	0.4128	1410.47339	44.23561	7.0901
2	13.334	VV	0.5157	1.84830e4	477.47458	92.9099

sum: 1.98934e4 521.71019

Compound 10

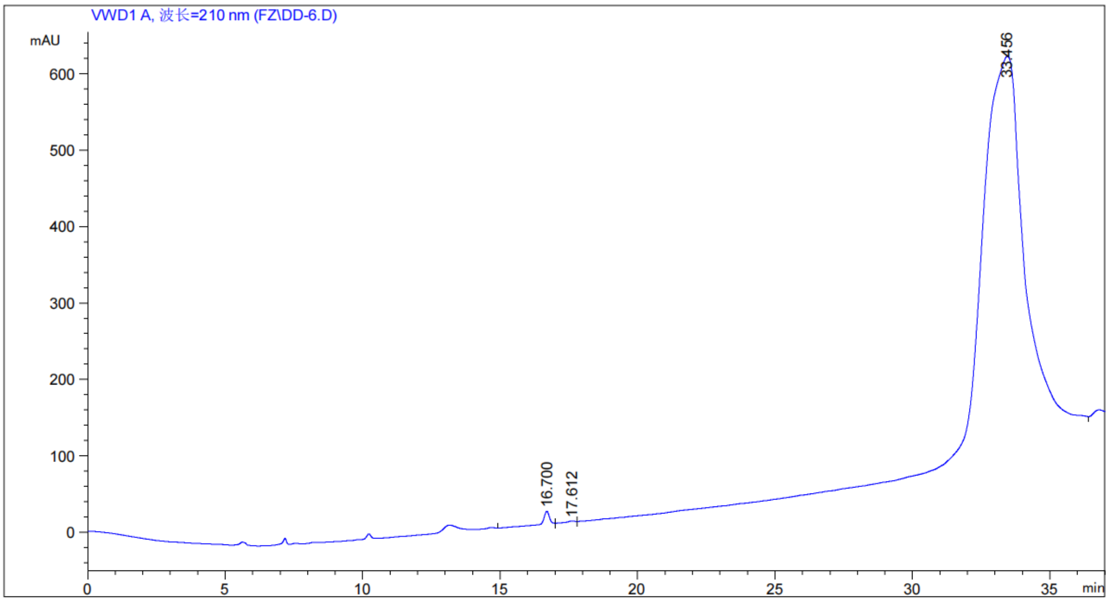

Peak Retention time Peak type Peak width Peak area Peak height Peak area%

#	[min]		[min]	mAU *s	[mAU]	%
1	16.700	VV	0.9569	3466.27905	44.70897	2.3467
2	17.612	VV	0.5739	1434.41748	32.08900	0.9711
3	33.456	VV	2.9919	1.42808e5	638.95795	96.6822

sum : 1.47709e5 715.75591

Compound 11

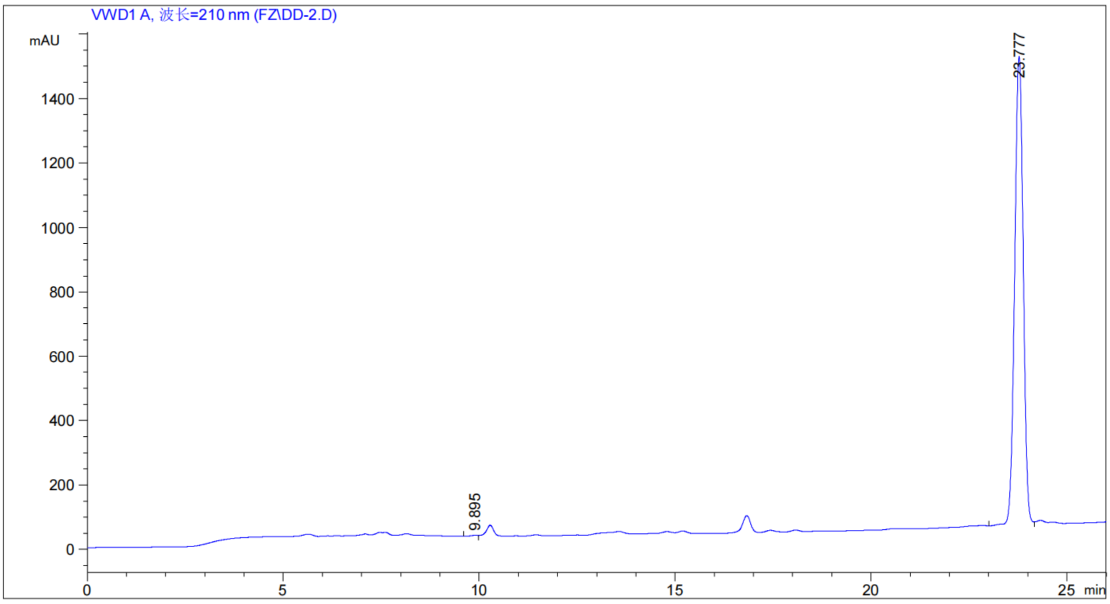

Peak Retention time Peak type Peak width Peak area Peak height Peak area%

```
  #    [min]          [min]  mAU    *s    [mAU    ]        %
----|-------|----|-------|----------|----------|--------|
   1    9.895 VV    0.2970  797.91742   36.32996   2.8869
   2   23.777 VV    0.2641 2.68411e4  1520.83435  97.1131

sum :                     2.76390e4  1557.16431
```

Table S1. Gibbs free energies[a] and equilibrium populations[b] of low-energy conformers of phaseolorin J (**1**).

Conformers	In gas	
	G^a	P (%)[b]
**1.-1**	-694991.07437097	49.68
**1-2**	-694991.07499848	49.74
**1-3**	-694988.43757395	0.58

[a]B3LYP/6-31G(d,p), in kcal/mol. [b]From G values at 298.15K.

Table S2. Cartesian coordinates for the low-energy reoptimized MMFF conformers of phaseolorin J (**1**) at B3LYP/6-31G(d,p) level of theory in gas.

Conformer **1-1**

**1-1**		Standard Orientation (Ångstroms)			
Center number	Atomic number	Atomic Type	X	Y	Z
1.	6.	0.	-0.259242	4.493108	-0.793624
2.	6.	0.	-0.744437	4.403127	0.505704
3.	6.	0.	-0.861031	3.178465	1.174015
4.	6.	0.	-0.494533	2.016384	0.508435
5.	6.	0.	-0.019896	2.062035	-0.824450
6.	6.	0.	0.115809	3.323391	-1.465967
7.	8.	0.	-0.612927	0.839636	1.199328
8.	6.	0.	0.055840	-0.342818	0.684149
9.	6.	0.	-0.194352	-0.445422	-0.833875
10.	6.	0.	0.292864	0.835299	-1.524879
11.	6.	0.	-0.624762	-1.529121	1.397156
12.	6.	0.	-0.297981	-2.853846	0.730658
13.	6.	0.	0.189366	-2.915321	-0.516675
14.	6.	0.	0.416679	-1.736661	-1.424117
15.	8.	0.	0.802279	0.795186	-2.658322
16.	8.	0.	0.585510	3.417113	-2.717735
17.	8.	0.	1.822916	-1.631696	-1.716719
18.	6.	0.	-0.583222	-4.077975	1.558167
19.	8.	0.	-2.055587	-1.380578	1.411593
20.	6.	0.	1.551409	-0.317619	1.101180
21.	8.	0.	2.421043	0.300686	0.181051

22.	8.	0.	-1.591195	-0.445138	-1.124655
23.	1.	0.	-0.156713	5.444461	-1.302309
24.	1.	0.	-1.028654	5.312703	1.027000
25.	1.	0.	-1.213818	3.119808	2.196989
26.	1.	0.	-0.264196	-1.557993	2.437891
27.	1.	0.	0.433211	-3.884968	-0.945469
28.	1.	0.	-0.125185	-1.908151	-2.362600
29.	1.	0.	0.812584	2.504720	-3.016365
30.	1.	0.	1.864837	-1.012018	-2.466403
31.	1.	0.	0.006012	-4.075056	2.483938
32.	1.	0.	-0.357236	-4.995809	1.010329
33.	1.	0.	-1.638514	-4.099868	1.853012
34.	1.	0.	-2.224510	-0.501552	1.788193
35.	1.	0.	1.869644	-1.347833	1.310348
36.	1.	0.	1.602348	0.244268	2.039205
37.	1.	0.	2.563041	-0.361688	-0.521470
38.	1.	0.	-2.056403	-0.869259	-0.378706

Conformer **1-2**

**1-2**		Standard Orientation (Ångstroms)			
Center number	Atom number	Type	X	Y	Z
1.	6.	0.	-0.293088	4.487390	-0.814041
2.	6.	0.	-0.793413	4.397416	0.479512
3.	6.	0.	-0.906486	3.174126	1.150909
4.	6.	0.	-0.520636	2.013221	0.494270
5.	6.	0.	-0.029953	2.058589	-0.832859
6.	6.	0.	0.101517	3.318911	-1.477301
7.	8.	0.	-0.636209	0.837981	1.188119
8.	6.	0.	0.050581	-0.339653	0.685814
9.	6.	0.	-0.179934	-0.450414	-0.834751
10.	6.	0.	0.303351	0.832439	-1.524701
11.	6.	0.	-0.626949	-1.529840	1.395109
12.	6.	0.	-0.278930	-2.853880	0.738012
13.	6.	0.	0.223960	-2.915262	-0.503149
14.	6.	0.	0.450835	-1.737963	-1.412430
15.	8.	0.	0.827269	0.792985	-2.651512
16.	8.	0.	0.585711	3.412783	-2.723524
17.	8.	0.	1.859371	-1.620485	-1.688415
18.	6.	0.	-0.562000	-4.077611	1.566807
19.	8.	0.	-2.059226	-1.395569	1.391258
20.	6.	0.	1.540744	-0.297918	1.120811
21.	8.	0.	2.415447	0.324612	0.208428

22.	8.	0.	-1.573028	-0.464727	-1.142612
23.	1.	0.	-0.193428	5.437820	-1.325009
24.	1.	0.	-1.092794	5.306103	0.993864
25.	1.	0.	-1.271326	3.115709	2.169653
26.	1.	0.	-0.279021	-1.550840	2.440323
27.	1.	0.	0.482602	-3.884020	-0.925274
28.	1.	0.	-0.077997	-1.918336	-2.356684
29.	1.	0.	0.825217	2.501712	-3.016159
30.	1.	0.	1.904390	-1.002902	-2.439694
31.	1.	0.	0.015996	-4.065278	2.499554
32.	1.	0.	-0.320360	-4.995204	1.025294
33.	1.	0.	-1.620491	-4.108827	1.849075
34.	1.	0.	-2.241839	-0.517271	1.763113
35.	1.	0.	1.866361	-1.324014	1.338771
36.	1.	0.	1.574869	0.268807	2.056687
37.	1.	0.	2.572225	-0.339296	-0.489563
38.	1.	0.	-2.043334	-0.890296	-0.400705

Conformer **1-3**

**1-3**		Standard Orientation (Ångstroms)			
Center number	Atom number	Type	X	Y	Z
1.	6.	0.	-0.121899	4.487576	-0.855731
2.	6.	0.	-0.573605	4.408048	0.457698
3.	6.	0.	-0.731157	3.185861	1.119595
4.	6.	0.	-0.444353	2.010971	0.435121
5.	6.	0.	-0.003100	2.046264	-0.913335
6.	6.	0.	0.173774	3.309080	-1.549323
7.	8.	0.	-0.596634	0.838947	1.113473
8.	6.	0.	0.043275	-0.348625	0.576366
9.	6.	0.	-0.265797	-0.457575	-0.932110
10.	6.	0.	0.222469	0.813936	-1.638521
11.	6.	0.	-0.615624	-1.531785	1.320705
12.	6.	0.	-0.334883	-2.859973	0.645530
13.	6.	0.	0.086042	-2.931269	-0.624341
14.	6.	0.	0.318317	-1.755281	-1.536084
15.	8.	0.	0.702494	0.771336	-2.785582
16.	8.	0.	0.609876	3.390904	-2.813457
17.	8.	0.	1.718686	-1.671309	-1.806559
18.	6.	0.	-0.599083	-4.077702	1.489522
19.	8.	0.	-2.049495	-1.367002	1.370930
20.	6.	0.	1.548387	-0.283201	0.890515
21.	8.	0.	1.694669	-0.192401	2.303348

22.	8.	0.	-1.670776	-0.430096	-1.166635
23.	1.	0.	0.010926	5.438091	-1.358976
24.	1.	0.	-0.796754	5.325840	0.994203
25.	1.	0.	-1.053153	3.133602	2.152809
26.	1.	0.	-0.222470	-1.546822	2.343386
27.	1.	0.	0.295503	-3.904863	-1.062521
28.	1.	0.	-0.237139	-1.915952	-2.470388
29.	1.	0.	0.768812	2.468898	-3.130041
30.	1.	0.	1.800653	-1.011949	-2.514528
31.	1.	0.	0.036219	-4.082477	2.384153
32.	1.	0.	-0.417002	-5.000218	0.932892
33.	1.	0.	-1.638431	-4.081123	1.837231
34.	1.	0.	-2.203267	-0.517373	1.813210
35.	1.	0.	1.982682	0.594570	0.391910
36.	1.	0.	2.036775	-1.169670	0.475923
37.	1.	0.	2.636848	-0.252592	2.502546
38.	1.	0.	-2.111787	-0.832665	-0.392935

Table S3. Gibbs free energies[a] and equilibrium populations[b] of low-energy conformers of phomoparagin D (**5**).

Conformers	In gas	
	G^a	P (%)[b]
**5-1**	-953546.56610655	92.90
**5-2**	-953544.26251734	1.90
**5-3**	-953544.85990686	5.20

[a]B3LYP/6-31G(d,p), in kcal/mol. [b]From G values at 298.15K.

Table S4. Cartesian coordinates for the low-energy reoptimized MMFF conformers of phomoparagin D (**5**) at B3LYP/6-31G(d,p) level of theory in gas.

Conformer **5-1**

**5-1**		Standard Orientation (Ångstroms)			
Center number	Atomic number	Atomic Type	X	Y	Z
1.	6.	0.	-1.604190	2.227710	1.437805
2.	6.	0.	-1.530347	0.707112	1.170235
3.	6.	0.	-0.176412	0.246035	0.511293
4.	6.	0.	0.611138	1.468136	-0.079859
5.	6.	0.	-0.357283	2.455913	-0.795094
6.	6.	0.	-1.433014	2.995226	0.141234
7.	6.	0.	-2.137023	4.076462	-0.202232
8.	8.	0.	0.346427	3.565082	-1.335655
9.	6.	0.	-2.839407	2.623964	2.253757
10.	6.	0.	-2.677734	0.173093	0.257223
11.	7.	0.	-1.978392	-0.680452	-0.691083

12.	6.	0.	-0.625850	-0.728396	-0.598433
13.	6.	0.	0.649254	-0.529942	1.579284
14.	6.	0.	-3.773223	-0.605369	1.021452
15.	6.	0.	-4.849648	-1.164309	0.115134
16.	6.	0.	-5.894631	-0.349180	-0.344540
17.	6.	0.	-6.864191	-0.849438	-1.212744
18.	6.	0.	-6.806241	-2.178137	-1.638825
19.	6.	0.	-5.774287	-3.001321	-1.188262
20.	6.	0.	-4.805219	-2.496850	-0.318425
21.	8.	0.	1.100051	0.403034	2.545376
22.	8.	0.	0.102659	-1.433673	-1.290748
23.	1.	0.	-1.614314	0.209122	2.141758
24.	1.	0.	1.041597	2.002082	0.772310
25.	6.	0.	1.798577	-1.430759	1.126679
26.	6.	0.	3.204378	-1.025410	1.084187
27.	6.	0.	1.738981	1.114006	-1.018016
28.	6.	0.	2.965156	1.643969	-0.916075
29.	6.	0.	4.146325	1.326251	-1.789136
30.	6.	0.	4.283717	-1.761132	0.288387
31.	6.	0.	4.502097	-1.119227	-1.094755
32.	6.	0.	5.111784	0.309961	-1.120906
33.	6.	0.	6.477331	0.309334	-1.823581
34.	8.	0.	5.520071	-1.613440	1.008781
35.	6.	0.	3.968592	-3.254635	0.121488
36.	8.	0.	2.685037	-1.742113	2.239478
37.	1.	0.	-0.728449	2.447765	2.065013
38.	1.	0.	-0.851530	1.900464	-1.613609
39.	1.	0.	-1.930363	4.597907	-1.130388
40.	1.	0.	-2.912495	4.491413	0.432646
41.	1.	0.	1.117907	3.203275	-1.798562
42.	1.	0.	-2.909677	2.017836	3.162687
43.	1.	0.	-3.767025	2.497985	1.685053
44.	1.	0.	-2.784472	3.672179	2.560697
45.	1.	0.	-3.154818	1.005721	-0.277461
46.	1.	0.	-2.460974	-1.243900	-1.378248
47.	1.	0.	-0.064494	-1.230528	2.051987
48.	1.	0.	-3.294329	-1.417709	1.580321
49.	1.	0.	-4.218242	0.073207	1.757272
50.	1.	0.	-5.951315	0.685173	-0.013722
51.	1.	0.	-7.667920	-0.203246	-1.553744
52.	1.	0.	-7.562865	-2.569005	-2.312578
53.	1.	0.	-5.724236	-4.038071	-1.508310
54.	1.	0.	-4.010025	-3.147167	0.037783

55.	1.	0.	1.734660	-0.090650	3.092474
56.	1.	0.	1.489361	-2.292159	0.544018
57.	1.	0.	3.439998	0.012678	1.305206
58.	1.	0.	1.510233	0.428437	-1.831384
59.	1.	0.	3.169125	2.312489	-0.077083
60.	1.	0.	4.699493	2.251029	-1.999115
61.	1.	0.	3.816814	0.926789	-2.756767
62.	1.	0.	5.163162	-1.794274	-1.650450
63.	1.	0.	3.537723	-1.140029	-1.613643
64.	1.	0.	5.284958	0.634229	-0.087898
65.	1.	0.	7.160214	-0.387735	-1.328802
66.	1.	0.	6.382341	0.002208	-2.872577
67.	1.	0.	6.935943	1.304159	-1.807335
68.	1.	0.	5.384389	-2.003852	1.883861
69.	1.	0.	3.708898	-3.706814	1.083058
70.	1.	0.	4.854513	-3.755939	-0.275906
71.	1.	0.	3.139222	-3.421431	-0.572491

Conformer **5-2**

**5-2**		Standard Orientation (Ångstroms)			
Center number	Atom number	Type	X	Y	Z
1.	6.	0.	2.133490	1.557655	-0.902245
2.	6.	0.	1.767212	0.261667	-0.126510
3.	6.	0.	0.232245	0.167813	0.239773
4.	6.	0.	-0.550588	1.428748	-0.256781
5.	6.	0.	0.154110	2.744822	0.186772
6.	6.	0.	1.646654	2.752177	-0.117698
7.	6.	0.	2.413685	3.758050	0.308711
8.	8.	0.	-0.424440	3.859449	-0.487721
9.	6.	0.	3.599551	1.612853	-1.337582
10.	6.	0.	2.545381	0.097406	1.220071
11.	7.	0.	1.492670	0.149780	2.224516
12.	6.	0.	0.212657	0.085350	1.778240
13.	6.	0.	-0.314910	-1.162235	-0.354639
14.	6.	0.	3.348176	-1.224322	1.355302
15.	6.	0.	4.578081	-1.312423	0.479601
16.	6.	0.	4.574277	-2.056616	-0.707282
17.	6.	0.	5.707743	-2.115597	-1.520601
18.	6.	0.	6.867288	-1.430844	-1.156719
19.	6.	0.	6.888473	-0.692982	0.028693
20.	6.	0.	5.754474	-0.637845	0.838107
21.	8.	0.	-0.339943	-1.029106	-1.764943

22.	8.	0.	-0.779204	-0.013468	2.494317
23.	1.	0.	2.018283	-0.573425	-0.785271
24.	1.	0.	-0.520485	1.403196	-1.349907
25.	6.	0.	-1.638636	-1.725951	0.157031
26.	6.	0.	-2.918151	-1.572078	-0.537413
27.	6.	0.	-1.998087	1.521636	0.160195
28.	6.	0.	-3.000217	1.750651	-0.699018
29.	6.	0.	-4.455168	1.894298	-0.342172
30.	6.	0.	-4.286668	-1.787617	0.108111
31.	6.	0.	-4.911745	-0.460174	0.572343
32.	6.	0.	-5.261812	0.580373	-0.527597
33.	6.	0.	-6.770423	0.868092	-0.546727
34.	8.	0.	-5.156321	-2.310561	-0.911775
35.	6.	0.	-4.230460	-2.774216	1.282790
36.	8.	0.	-2.124806	-2.776759	-0.726133
37.	1.	0.	1.539173	1.515994	-1.825547
38.	1.	0.	0.014834	2.857121	1.275400
39.	1.	0.	1.988770	4.581622	0.874896
40.	1.	0.	3.473735	3.816232	0.085264
41.	1.	0.	-1.385582	3.763409	-0.393376
42.	1.	0.	3.860216	0.728428	-1.925510
43.	1.	0.	4.292889	1.658124	-0.492749
44.	1.	0.	3.774575	2.496908	-1.958068
45.	1.	0.	3.229328	0.941064	1.368979
46.	1.	0.	1.675135	0.075217	3.216257
47.	1.	0.	0.424032	-1.936820	-0.074333
48.	1.	0.	3.649971	-1.322051	2.406620
49.	1.	0.	2.673188	-2.062403	1.147355
50.	1.	0.	3.678938	-2.602686	-0.993438
51.	1.	0.	5.684721	-2.700710	-2.435421
52.	1.	0.	7.750379	-1.477663	-1.786935
53.	1.	0.	7.790148	-0.165104	0.325636
54.	1.	0.	5.783546	-0.068962	1.764699
55.	1.	0.	-0.780513	-1.834041	-2.085060
56.	1.	0.	-1.643108	-1.986991	1.210956
57.	1.	0.	-2.935841	-0.937927	-1.419789
58.	1.	0.	-2.197524	1.467556	1.228483
59.	1.	0.	-2.770653	1.775154	-1.766316
60.	1.	0.	-4.901748	2.672385	-0.974869
61.	1.	0.	-4.563209	2.232975	0.696232
62.	1.	0.	-5.827778	-0.724149	1.113327
63.	1.	0.	-4.233963	-0.024828	1.314505
64.	1.	0.	-5.006132	0.156829	-1.506055

65.	1.	0.	-7.034035	1.564905	-1.349933
66.	1.	0.	-7.333917	-0.057036	-0.700909
67.	1.	0.	-7.100769	1.311022	0.401087
68.	1.	0.	-4.757123	-3.134392	-1.225885
69.	1.	0.	-5.251897	-3.026511	1.578197
70.	1.	0.	-3.710939	-3.692410	0.993527
71.	1.	0.	-3.717417	-2.347091	2.149835

Conformer **5-3**

**5-3**		Standard Orientation (Ångstroms)			
Center number	Atom number	Type	X	Y	Z
1.	6.	0.	2.141018	2.280959	-1.153508
2.	6.	0.	2.058758	0.951238	-0.368058
3.	6.	0.	0.591100	0.546251	0.040702
4.	6.	0.	-0.399253	1.751291	-0.139913
5.	6.	0.	0.239098	3.075465	0.372898
6.	6.	0.	1.546956	3.409818	-0.336020
7.	6.	0.	2.079942	4.628075	-0.215074
8.	8.	0.	-0.644848	4.173818	0.194015
9.	6.	0.	3.547076	2.557259	-1.696075
10.	6.	0.	2.908235	0.948928	0.941410
11.	7.	0.	1.980159	0.438993	1.938270
12.	6.	0.	0.719620	0.149041	1.524559
13.	6.	0.	0.179268	-0.695118	-0.803377
14.	6.	0.	4.224777	0.125020	0.887364
15.	6.	0.	4.089266	-1.328371	0.487629
16.	6.	0.	3.669733	-2.299783	1.408940
17.	6.	0.	3.528699	-3.635126	1.031392
18.	6.	0.	3.811901	-4.028217	-0.277887
19.	6.	0.	4.242589	-3.077082	-1.202994
20.	6.	0.	4.380652	-1.741806	-0.820580
21.	8.	0.	-0.005311	-0.263877	-2.142259
22.	8.	0.	-0.156817	-0.333768	2.234949
23.	1.	0.	2.443311	0.169177	-1.026748
24.	1.	0.	-0.557030	1.863129	-1.216580
25.	6.	0.	-1.002895	-1.549606	-0.347946
26.	6.	0.	-2.371516	-1.405733	-0.846989
27.	6.	0.	-1.752120	1.581751	0.507316
28.	6.	0.	-2.905690	1.805366	-0.135899
29.	6.	0.	-4.285361	1.643969	0.438105
30.	6.	0.	-3.606044	-1.966702	-0.140020
31.	6.	0.	-4.283600	-0.904965	0.746451

32.	6.	0.	-4.945856	0.301131	0.024227
33.	6.	0.	-6.458272	0.333208	0.289465
34.	8.	0.	-4.558718	-2.317875	-1.159645
35.	6.	0.	-3.281863	-3.210696	0.699733
36.	8.	0.	-1.482012	-2.399114	-1.429319
37.	1.	0.	1.488904	2.133914	-2.026183
38.	1.	0.	0.450169	2.945131	1.450346
39.	1.	0.	1.575125	5.397461	0.359153
40.	1.	0.	3.008036	4.907841	-0.701896
41.	1.	0.	-1.514522	3.881731	0.508645
42.	1.	0.	3.910950	1.700632	-2.272654
43.	1.	0.	4.266091	2.761114	-0.895212
44.	1.	0.	3.545109	3.424588	-2.362274
45.	1.	0.	3.190352	1.978731	1.200193
46.	1.	0.	2.232457	0.283295	2.904538
47.	1.	0.	1.039912	-1.385191	-0.755792
48.	1.	0.	4.901088	0.635777	0.194101
49.	1.	0.	4.696328	0.192747	1.876833
50.	1.	0.	3.455906	-2.010695	2.433940
51.	1.	0.	3.202319	-4.369498	1.762049
52.	1.	0.	3.704999	-5.068127	-0.571672
53.	1.	0.	4.476072	-3.372988	-2.221627
54.	1.	0.	4.728543	-1.010122	-1.545613
55.	1.	0.	-0.394526	-1.028643	-2.599002
56.	1.	0.	-0.845112	-2.069042	0.591643
57.	1.	0.	-2.579390	-0.580992	-1.523851
58.	1.	0.	-1.764648	1.309065	1.560582
59.	1.	0.	-2.864692	2.056637	-1.197779
60.	1.	0.	-4.919798	2.469435	0.089322
61.	1.	0.	-4.257012	1.706780	1.533474
62.	1.	0.	-5.049173	-1.430202	1.329156
63.	1.	0.	-3.533048	-0.562100	1.466914
64.	1.	0.	-4.815146	0.175808	-1.057120
65.	1.	0.	-6.941827	1.155395	-0.249493
66.	1.	0.	-6.921961	-0.603608	-0.033839
67.	1.	0.	-6.669621	0.463829	1.358048
68.	1.	0.	-4.130188	-2.971672	-1.730192
69.	1.	0.	-2.704498	-2.959708	1.594755
70.	1.	0.	-4.220312	-3.673331	1.014996
71.	1.	0.	-2.709952	-3.936108	0.113903

Table S5. The calculated ^{13}C NMR data for isomers of phaseolorin J (**1**)

no.	*δ*exp	δcal		δscal		corrected error	
		**1**	8-*epi*-**1**	**1**	8-*epi*-**1**	**1**	8-*epi*-**1**
1	163.2	173.0	173.0	165.7	165.3	2.5	2.1
2	109.3	114.8	113.2	108.6	107.3	-0.7	-2.0
3	138.9	144.0	145.2	137.3	138.3	-1.6	-0.6
4	108.7	109.2	110.3	103.2	104.5	-5.5	-4.2
4a	160.8	167.4	169.1	160.3	161.6	-0.5	0.8
5	74.3	78.0	76.7	72.5	71.7	-1.8	-2.6
6	140.5	146.6	153.0	139.8	146.0	-0.7	5.5
7	122.3	133.9	127.5	127.4	121.1	5.1	-1.2
8	67.9	76.7	74.4	71.2	69.6	3.3	1.7
8a	74.5	77.5	77.9	72.0	72.9	-2.5	-1.6
9	197.2	205.4	205.0	197.6	196.4	0.4	-0.8
9a	108.4	113.1	111.2	106.9	105.2	-1.5	-3.2
10a	86.5	93.0	91.8	87.2	86.4	0.7	-0.1
11	19.2	24.0	23.2	19.5	19.7	0.3	0.5
12	64.1	72.0	74.7	66.6	69.8	2.5	5.7

Table S6. DFT-optimized structures and thermodynamic parameters for low-energy conformers of **1.**

Conformers	Conf. A	Conf. B	Conf. C
DFT-optimized structures	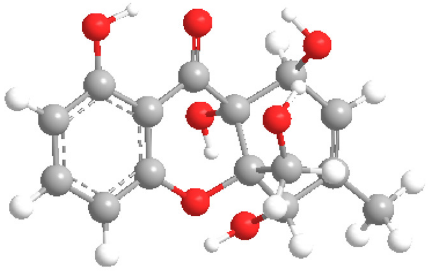	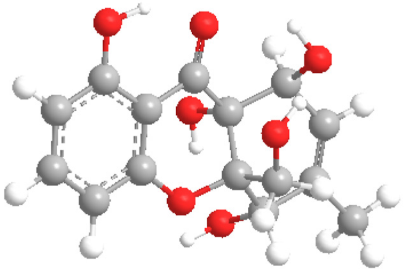	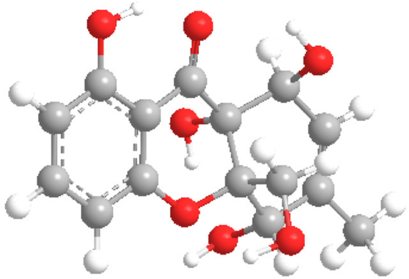
Population	48.53%	48.53%	2.94%
Total energy (a.u.)	-1107.75139664	-1107.75139657	-1107.74875446
Sum of electronic and zero-point energies (a.u.)	-1107.449975	-1107.449976	-1107.448173
Sum of electronic and thermal energies (a.u.)	-1107.431472	-1107.431474	-1107.429249
Sum of electronic and thermal enthalpies (a.u.)	-1107.430528	-1107.430529	-1107.428305
Sum of electronic and thermal free energies (a.u.)	-1107.494222	-1107.494223	-1107.492928

Table S7. DFT-optimized structures and thermodynamic parameters for low-energy conformers of 8-*epi*-**1.**

Conformers	Conf. A	Conf. B
DFT-optimized structures	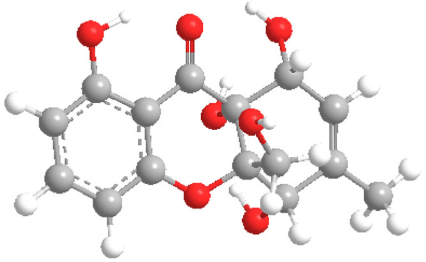	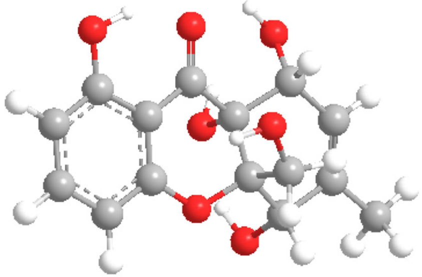
Population	5.13%	94.87%
Total energy (a.u.)	-1107.74778988	-1107.75054174
Sum of electronic and zero-point energies (a.u.)	-1107.447238	-1107.449580
Sum of electronic and thermal energies (a.u.)	-1107.428344	-1107.430830
Sum of electronic and thermal enthalpies (a.u.)	-1107.427399	-1107.429886
Sum of electronic and thermal free energies (a.u.)	-1107.492019	-1107.494259

Table S8. Optimized Z-matrixes of **1** in the gas phase (Å) at B3LYP/6-31G(d) level.

Conf. A				Conf. B				Conf. C			
C	-4.236	-0.536	-0.08487	C	-4.23596	-0.53598	-0.08464	C	-4.26519	-0.45983	-0.12029
C	-3.84304	-1.84472	0.170022	C	-3.84294	-1.8447	0.170173	C	-3.84994	-1.7849	-0.18463
C	-2.49582	-2.20659	0.290116	C	-2.49574	-2.20661	0.290019	C	-2.49589	-2.14042	-0.18446
C	-1.52385	-1.22766	0.132729	C	-1.52374	-1.22773	0.132434	C	-1.53889	-1.13437	-0.13828
C	-1.88083	0.112434	-0.15322	C	-1.88076	0.112417	-0.15341	C	-1.91842	0.230954	-0.09686
C	-3.25813	0.4543	-0.2391	C	-3.25809	0.454311	-0.239	C	-3.30476	0.557482	-0.06896
O	-0.21869	-1.62135	0.268466	O	-0.2186	-1.62138	0.268002	O	-0.22678	-1.52528	-0.11096
C	0.802458	-0.60333	0.444643	C	0.802407	-0.60326	0.444549	C	0.782259	-0.53818	0.268063
C	0.562565	0.533509	-0.56876	C	0.562588	0.533449	-0.56902	C	0.509271	0.790094	-0.45978
C	-0.85007	1.103151	-0.38036	C	-0.8501	1.1031	-0.38072	C	-0.90859	1.271072	-0.12938
C	2.132758	-1.31123	0.115701	C	2.132726	-1.31114	0.115972	C	2.123004	-1.12603	-0.21926
C	3.265598	-0.32152	-0.0903	C	3.265602	-0.32144	-0.09012	C	3.234003	-0.09474	-0.20769
C	3.032842	0.975997	-0.33485	C	3.032825	0.976	-0.33511	C	2.972633	1.218379	-0.1628
C	1.675099	1.604506	-0.49572	C	1.675083	1.604484	-0.49588	C	1.598907	1.834815	-0.12664
O	-1.07485	2.315122	-0.54089	O	-1.07482	2.31511	-0.541	O	-1.16498	2.478326	0.01313
O	-3.6499	1.714366	-0.48215	O	-3.64981	1.714406	-0.4819	O	-3.71848	1.830515	-0.00611
O	1.478038	2.574437	0.551933	O	1.478089	2.57422	0.552011	O	1.418231	2.438339	1.155091
C	4.652054	-0.90465	-0.02333	C	4.652078	-0.90445	-0.02282	C	4.630995	-0.65112	-0.28202
O	2.018287	-2.11072	-1.07617	O	2.018449	-2.11082	-1.0758	O	2.008362	-1.62284	-1.57174
C	0.84154	-0.15703	1.932192	C	0.841086	-0.15691	1.932195	C	0.763921	-0.44914	1.811867
O	0.521085	0.029645	-1.90394	O	0.521166	0.029345	-1.90411	O	0.453341	0.601643	-1.87257
O	0.022654	0.94536	2.249675	O	0.022367	0.945573	2.249538	O	0.946475	-1.72771	2.398344
H	-5.28065	-0.25606	-0.16276	H	-5.28061	-0.25604	-0.16235	H	-5.31485	-0.18845	-0.10683
H	-4.60421	-2.61015	0.295725	H	-4.60412	-2.61012	0.296003	H	-4.59814	-2.5721	-0.22145
H	-2.19799	-3.22421	0.517124	H	-2.19785	-3.22423	0.517003	H	-2.18093	-3.17794	-0.21149
H	2.388146	-1.97563	0.95688	H	2.387955	-1.9754	0.957309	H	2.385955	-1.95964	0.443533
H	3.874837	1.655987	-0.45115	H	3.874782	1.656012	-0.45158	H	3.798475	1.926755	-0.13297
H	1.641332	2.125118	-1.46105	H	1.641177	2.125264	-1.46112	H	1.528411	2.59646	-0.91573
H	-2.83627	2.272762	-0.54051	H	-2.83615	2.272754	-0.54055	H	-2.91318	2.404623	0.033331
H	0.693907	3.080239	0.26081	H	0.693788	3.079955	0.261221	H	0.604307	2.967516	1.075208

H	5.417269	-0.14552	-0.20888	H	5.417254	-0.14528	-0.20835	H	5.379463	0.146083	-0.30954
H	4.845172	-1.35494	0.959807	H	4.845009	-1.35455	0.960443	H	4.844796	-1.29508	0.581523
H	4.767431	-1.70272	-0.76705	H	4.767666	-1.70267	-0.76635	H	4.750353	-1.27002	-1.17989
H	1.242266	-2.68299	-0.93231	H	1.242329	-2.68302	-0.93221	H	1.287576	-2.27703	-1.5565
H	0.493715	-1.00945	2.524419	H	0.492854	-1.00928	2.524254	H	1.575948	0.187022	2.161666
H	1.886385	0.040486	2.2094	H	1.885918	0.04029	2.209743	H	-0.17784	0.009213	2.145197
H	1.119196	-0.74507	-1.95315	H	1.119601	-0.74514	-1.95331	H	1.06526	-0.13024	-2.10096
H	0.513357	1.730239	1.927883	H	0.513248	1.730504	1.928138	H	0.235134	-2.29554	2.059964

Table S9. Optimized Z-matrixes of 8-*epi*-**1** in the gas phase (Å) at B3LYP/6-31G(d) level.

Conf. A				Conf. B			
C	-4.24481	-0.45096	-0.28011	C	-4.2409	-0.44291	-0.28124
C	-3.86676	-1.79076	-0.31712	C	-3.86501	-1.78265	-0.33534
C	-2.53077	-2.19149	-0.22921	C	-2.52962	-2.18771	-0.25391
C	-1.54238	-1.21935	-0.10723	C	-1.53747	-1.21914	-0.123
C	-1.885	0.157512	-0.09079	C	-1.87927	0.160176	-0.08143
C	-3.25751	0.531412	-0.1637	C	-3.25233	0.537586	-0.15474
O	-0.25919	-1.66102	-0.02484	O	-0.25605	-1.66083	-0.04184
C	0.781576	-0.7587	0.398447	C	0.786417	-0.75471	0.391053
C	0.583013	0.626246	-0.22963	C	0.591087	0.628849	-0.23585
C	-0.84367	1.151475	-0.03564	C	-0.83278	1.155909	-0.03314
C	2.131968	-1.3545	-0.0952	C	2.135739	-1.35413	-0.09077
C	3.260604	-0.32764	0.018644	C	3.265942	-0.3317	0.037943
C	3.030654	0.984489	0.163291	C	3.03745	0.981313	0.177021
C	1.664686	1.610152	0.256607	C	1.672979	1.610912	0.256046
O	-1.05266	2.380358	0.021037	O	-1.04659	2.381895	0.028389
O	-3.62726	1.822037	-0.13306	O	-3.6198	1.826749	-0.11036
O	1.643478	2.75707	-0.6023	O	1.66143	2.754677	-0.60809
C	4.647077	-0.90002	-0.10682	C	4.652308	-0.90818	-0.06701
O	2.063305	-1.88616	-1.40365	O	2.077467	-1.88	-1.40346
C	0.810899	-0.73768	1.938124	C	0.787057	-0.72691	1.936937

O	0.711446	0.498817	-1.65946	O	0.714423	0.508522	-1.66569
O	-0.21896	0.105471	2.437997	O	-0.2115	0.093128	2.526701
H	-5.28385	-0.14661	-0.33959	H	-5.27934	-0.13632	-0.33795
H	-4.63646	-2.55283	-0.40815	H	-4.63611	-2.542	-0.43527
H	-2.24477	-3.237	-0.24545	H	-2.24543	-3.23329	-0.28697
H	2.371528	-2.21408	0.54588	H	2.365661	-2.21747	0.548511
H	3.859728	1.688987	0.200998	H	3.867635	1.683643	0.226016
H	1.43726	1.91707	1.28718	H	1.439807	1.923771	1.283547
H	-2.80418	2.363553	-0.04706	H	-2.79581	2.368781	-0.02925
H	0.771547	3.169408	-0.43534	H	0.801988	3.187311	-0.43338
H	5.410064	-0.1161	-0.12458	H	5.417352	-0.12624	-0.0799
H	4.872839	-1.58067	0.725934	H	4.865623	-1.58368	0.773038
H	4.724951	-1.4897	-1.02753	H	4.740146	-1.50365	-0.9831
H	1.687149	-1.16978	-1.94961	H	1.711236	-1.15953	-1.95059
H	0.674738	-1.77739	2.267296	H	0.706858	-1.76895	2.276215
H	1.798283	-0.39508	2.275914	H	1.741396	-0.32988	2.291042
H	1.060379	1.373332	-1.93538	H	1.066823	1.383337	-1.93766
H	-0.21796	0.053158	3.405746	H	-1.07249	-0.3391	2.414987
